# A Comprehensive Methodological Survey of Human Activity Recognition Across Diverse Data Modalities

**DOI:** 10.3390/s25134028

**Published:** 2025-06-27

**Authors:** Jungpil Shin, Najmul Hassan, Abu Saleh Musa Miah, Satoshi Nishimura

**Affiliations:** School of Computer Science and Engineering, The University of Aizu, Aizuwakamatsu 965-8580, Japan; d8252102@u-aizu.ac.jp (N.H.); musa@u-aizu.ac.jp (A.S.M.M.); nisim@u-aizu.ac.jp (S.N.)

**Keywords:** human activity recognition (HAR), diverse modality, deep learning (DL), machine learning (ML), vision and sensor based HAR, classification

## Abstract

Human Activity Recognition (HAR) systems aim to understand human behavior and assign a label to each action, attracting significant attention in computer vision due to their wide range of applications. HAR can leverage various data modalities, such as RGB images and video, skeleton, depth, infrared, point cloud, event stream, audio, acceleration, and radar signals. Each modality provides unique and complementary information suited to different application scenarios. Consequently, numerous studies have investigated diverse approaches for HAR using these modalities. This survey includes only peer-reviewed research papers published in English to ensure linguistic consistency and academic integrity. This paper presents a comprehensive survey of the latest advancements in HAR from 2014 to 2025, focusing on Machine Learning (ML) and Deep Learning (DL) approaches categorized by input data modalities. We review both single-modality and multi-modality techniques, highlighting fusion-based and co-learning frameworks. Additionally, we cover advancements in hand-crafted action features, methods for recognizing human–object interactions, and activity detection. Our survey includes a detailed dataset description for each modality, as well as a summary of the latest HAR systems, accompanied by a mathematical derivation for evaluating the deep learning model for each modality, and it also provides comparative results on benchmark datasets. Finally, we provide insightful observations and propose effective future research directions in HAR.

## 1. Introduction

Human Activity Recognition (HAR) has been a very active research topic for the past two decades in the field of computer vision and Artificial Intelligence (AI) that focuses on the automated analysis and understanding of human actions and recognition based on the movements and poses of the entire body.

### 1.1. Rationale

HAR plays an important role in various applications such as surveillance, healthcare [1,2,3], remote monitoring, intelligent human–machine interfaces, entertainment, storage video and retrieval [4,5], and human–computer interaction [6].

HAR is very important in computer vision and covers many research topics, including HAR in video, human tracking, and analysis and understanding in videos captured with a moving camera, where motion patterns exist due to video objects and the moving camera as well [7]. In such a scenario, it becomes ambiguous to recognize objects. The HAR methods were categorized into three distinct tiers: human action detection, human action tracking, and behavior understanding methods. In recent years, the investigation of interaction [8,9] and human action detection [10,11,12] has emerged as a prominent area of research. Many state-of-the-art techniques deal with action recognition using action frames as images and are only able to detect the presence of an object in them. They cannot properly recognize the object in an image or video. By properly recognizing an action in a video, it is possible to recognize the class of action more accurately. To perform action recognition, there has been an increased interest in this field in recent years due to the increased availability of computing resources as well as new advances in ML [13] and DL. Robust human action modelling and feature representation are essential components for achieving effective HAR. The main issue of representing and selecting features is a well-established problem within the fields of computer vision and ML [13]. Unlike the representation of features in an image domain, the representation of features of human actions in a video not only depicts the visual attributes of the human being(s) within the image domain but must also perform the extraction of alterations in visual attributes and pose. The problem of representation of features has been expanded from a 2D space to a 3D spatio-temporal context. In the past few years, many types of action representation techniques have been proposed. These techniques include various approaches, such as local and global features that rely on temporal and spatial alterations [14,15,16], trajectory features that are based on key point tracking [17], motion changes that are derived from depth information [18,19], and action features that are derived from human pose changes [20,21]. With the performance and successful application of DL to activity recognition and classification, many researchers have used DL for HAR. This facilitates the automatically learned features from the video dataset [22,23]. However, the aforementioned review articles have only examined certain specific facets, such as the Spatial Temporal Interest Point (STIP) and Histogram of Optical Flow (HOF)-found techniques for HAR, as well as approaches for analyzing human walking and DL-based techniques. Numerous novel approaches have been recently developed, primarily regarding the utilization of depth learning techniques for feature learning. Hence, a comprehensive examination of these fresh approaches for recognizing human actions is of significant interest. Additionally, HAR has critical applications in security and surveillance; this survey focuses on general-purpose recognition methods and does not cover security-specific techniques in depth.

### 1.2. Objective

Many researchers have been working to survey the HAR system article, which is mainly based on ML and DL techniques, with diverse feature extraction techniques. Such HAR literature was summarized by [24] within the framework of three key areas: sensor modality, deep models, and application. Vrigkas et al. [25] also reviewed HAR using RGB static images, covering both single-mode and multi-mode approaches. Vishwakarma et al. [26] summarized classical HAR methods, categorizing them into hierarchical and non-hierarchical methods based on feature representation. The survey by Ke et al. [27] provided a comprehensive overview of handcrafted methods in HAR. Additionally, surveys [28,29,30,31] extensively discuss the strengths and weaknesses of handcrafted versus DL methods, emphasizing the advantages of DL-based approaches. Xing et al. [32] focused on HAR development using 3D skeleton data, reviewing various DL-based techniques and comparing their performance across different dimensions. Presti et al. [33] presented HAR techniques based on 3D skeleton data. Methods for HAR using depth and skeleton data have been thoroughly reviewed by Ye et al. [19]; they also present HAR techniques using depth data.

Although certain review articles discuss data fusion methods, they offer a limited overview of HAR approaches to particular data types. Similarly, Subetha et al. [34] presented the same strategy to review action recognition methods. However, in distinction to those studies, we categorize HAR into five distinct categories: action recognition RGB and handcrafted features, action recognition RGB and DL, action recognition skeleton and handcrafted features, action recognition skeleton-based and DL, action recognition sensor-based methods, and action recognition using a multimodal dataset. The crucial element of the analysis regarding the literature on HAR is that most surveys have focused on the representations of human action features. The data of the image sequences that have been processed are typically well-segmented and consist solely of a single action event. More recently, many researchers have been working to conduct HAR survey studies with a specific point of view. For example, some researchers have surveyed Graph Convolutional Networks (GCNs) structures and data modalities for HAR and the application of GCNs in HAR [35,36]. Gupta et al. [37] explored current and future directions in skeleton-based HAR and introduced the skeleton-152 dataset, marking a significant advancement in the field. Meanwhile, Song et al. [38] reviewed advancements in human pose estimation and its applications in HAR, emphasizing its importance. Additionally, Shaikh et al. [39] focused on data integration and recognition approaches within a visual framework, specifically from an RGB-D perspective. Majumder et al. [40] and Wang et al. [41] provided reviews of popular methods using vision and inertial sensors for HAR. More recently, Wang et al. [42] surveyed HAR by performing two modalities of RGB-based and skeleton-based HAR techniques. Similarly, Sun et al. [43] surveyed HAR with various multi-modality methods.

#### 1.2.1. Research Gaps and New Research Challenges

Also, each survey paper can give us an overall summary of the existing work in this domain. Still, there is a lack of comparative studies of the various modalities, such as RGB, skeleton, sensor, and fusion-based and diverse modality-based HAR systems of recent technologies. From a data perspective, most reviews on HAR are limited to methodologies based on specific data, such as RGB, depth, and fusion data modalities. Moreover, we did not find a HAR survey paper that included diverse modality-based HAR, including their benchmark dataset and latest performance accuracy for 2014–2025. The studies of [8,42] inspired us to complete a survey study with current research trends for HAR.

#### 1.2.2. Our Contribution

**Figure 1:**
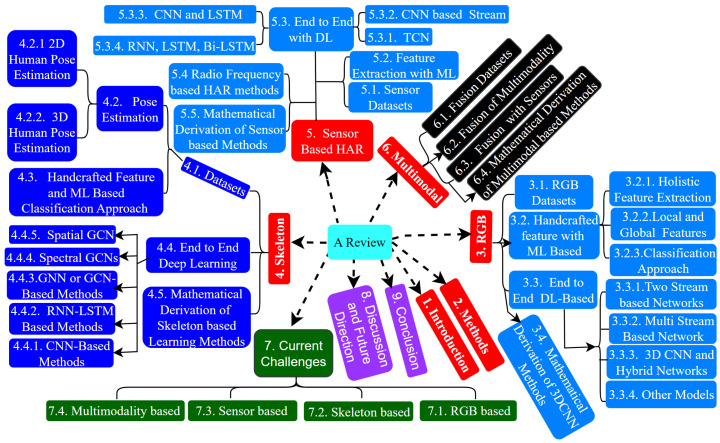
Diverse modality, including the structure of this paper.

Figure 1 demonstrates the proposed methodology flowchart. In this study, we survey state-of-the-art methods for HAR, addressing their challenges and future directions across vision-, sensor-, and fusion-based data modalities. We also summarize the current two-dimensions and three-dimensions pose estimation algorithms before discussing skeleton-based feature representation methods. Additionally, we categorize action recognition techniques into handcrafted feature-based ML and end-to-end DL-based methods. Our main contributions are as follows:

- **Comprehensive Review with Diverse Modality**: We conduct a thorough survey of RGB-based, skeleton-based, sensor-based, and fusion HAR-based methods, focusing on the evolution of data acquisition, environments, and human activity portrayals from 2014 to 2025.
- **Dataset Description**: We provide a detailed overview of benchmark public datasets for RGB, skeleton, sensor, and fusion data, highlighting their latest performance accuracy with references.
- **Unique Process**: Our study covers feature representation methods, common datasets, challenges, and future directions, emphasizing the extraction of distinguishable action features from video data despite environmental and hardware limitations. We also included the mathematical derivation for the evaluation of the deep learning model for each modality, such as from 3D CNN to Multi-View Transformer and GCN to EMS-TAGCN for pixel video and sequence of the skeleton dataset, respectively.
- **Identification of Gaps and Future Directions**: We identify significant gaps in the current research and propose future research directions supported by the latest performance data for each modality.
- **Evaluation of System Efficacy**: We assess existing HAR systems by analyzing their recognition accuracy and providing benchmark datasets for future development.
- **Guidance for Practitioners**: Our review offers practical guidance for developing robust and accurate HAR systems, providing insights into current techniques, highlighting challenges, and suggesting future research directions to advance HAR system development.

#### 1.2.3. Research Questions

This research addresses the following major questions:

1. What are the main difficulties faced in human activity recognition?
2. What are the major open challenges faced in human activity recognition?
3. What are the major algorithms involved in human activity recognition?

### 1.3. Organization of the Work

The paper is categorized as follows. The benchmark datasets are provided in Section 3.1. The action recognition RGB-data modality methods and skeleton data modality-based methods are discussed in Section 3 and Section 4, respectively. In Section 5, Section 6 and Section 7, we introduce sensor modality-based HAR, multimodal fusion modality-based, and current challenges, including four data modalities, respectively. We discuss future research trends and direction in Section 8. Finally, in Section 9, we summarized the conclusions. The detailed structure of this paper is shown in Figure 1.

## 2. Methods

This section outlines the methodology for conducting a comprehensive review of HAR research, focusing on studies published between 2014 and 2025 across diverse data modalities. The methodology comprises the article search protocol, eligibility criteria, article selection, quality appraisal, and data charting strategy.

### 2.1. Article Search Protocol

We performed an extensive search using databases such as IEEE Xplore, Scopus, Web of Science, SpringerLink, and ACM Digital Library. Boolean keyword combinations included the following:

- “Human Activity Recognition” OR “Human Action Recognition”
- “Computer Vision”, “RGB”, “Skeleton”, “Sensor”, “Multimodal”, “Deep Learning”, “Machine Learning”

### 2.2. Eligibility Criteria

To refine and ensure relevance in our initial search results, we applied the following criteria:

Inclusion Criteria:

- Publications from 2014 to 2025;
- Peer-reviewed journals, conference papers, book chapters, and lecture notes;
- Focus on HAR using RGB, skeleton, sensor, fusion HAR methods, or multimodal;
- Emphasis on the evolution of data acquisition, environments, and human activity portrayals.

Exclusion Criteria:

- Exclusion of studies lacking in-depth information about their experimental procedures;
- Exclusion of research articles where the complete text is not accessible, both in physical and digital formats;
- Exclusion of research articles that include opinions, keynote speeches, discussions, editorials, tutorials, remarks, introductions, viewpoints, and slide presentations.

### 2.3. Article Selection Process

The literature was screened through a multi-step process: title screening, abstract review, and full-text evaluation. We prioritized articles published in prestigious journals and conferences such as:

- IEEE Transactions on Pattern Analysis and Machine Intelligence (TPAMI);
- IEEE Transactions on Image Processing (TIP);
- International Conference on Computer Vision and Pattern Recognition (CVPR);
- IEEE International Conference on Computer Vision (ICCV);
- Springer, ELSEVIER, MDPI, Frontier, etc.

**Figure 2:**
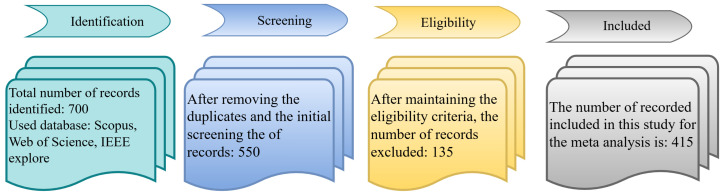
Article selection process block diagram.

**Figure 3:**
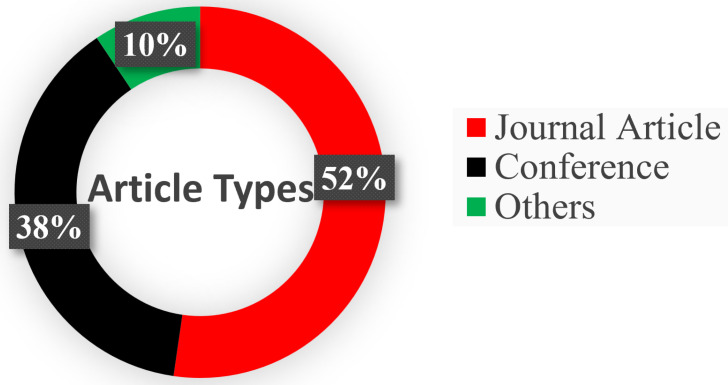
Distribution of article types across journal publications, conference proceedings, and other sources.

**Figure 4:**
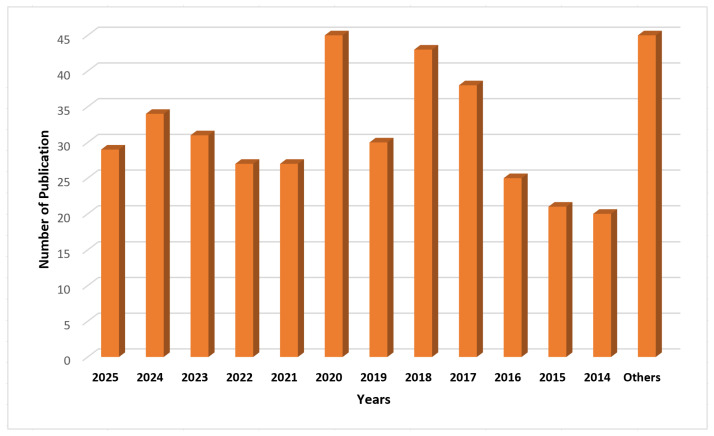
Year-wise distribution of selected HAR publications (2014–2025).

Figure 2 depicts the article selection process, while Figure 3 demonstrates the percentage of journals, conferences, and other ratios. Figure 4 shows the distribution of the year-wise number of references.

### 2.4. Critical Appraisal of Individual Sources

We reviewed each article through a structured process involving the following:

- Abstract review;
- Methodology analysis;
- Result evaluations;
- Discussion and conclusions.

We extracted key attributes from the selected studies using a structured data charting form. The extracted data included the following:

- Bibliographic information (author(s), publication year, and venue);
- Dataset characteristics and associated HAR modality (e.g., RGB, skeleton, sensor, fusion);
- Feature extraction techniques and classification models employed;
- Evaluation metrics and reported benchmark performance.

These data were synthesized and analyzed comparatively across modalities to identify trends, strengths, and methodological gaps in the literature.

## 3. RGB-Data Modality-Based Action Recognition Methods

**Figure 5:**
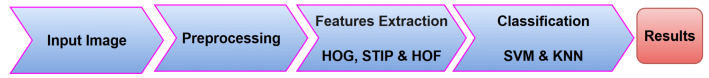
Workflow of RGB-based action recognition methods utilizing handcrafted features.

Figure 5 demonstrates a common workflow diagram of the RGB-based action recognition methods. The early stages of research regarding HAR were conducted based on the RGB data, and initially feature extraction mostly depended on manual annotation [44,45]. These annotations often relied on existing knowledge and prior assumptions. After this, DL-based architectures were developed to extract the most effective features and the best performance. The following sections describe the dataset, the methodological review of RGB-based handcrafted features with ML, and various ideas for DL-based approaches. Moreover, Table 1 lists detailed information about the RGB data modality, including the datasets, feature extraction methods, classifier, years, and performance accuracy.

### 3.1. RGB-Based Datasets of HAR

We provided the most popular benchmark HAR datasets, which come from the RGB skeleton, which is demonstrated in Table 2. The dataset table demonstrated the details of the datasets, including modalities, creation year, number of classes, number of subjects who participated in recording the dataset, number of samples, and the latest performance accuracy of the dataset with citations.

The RGB dataset encompasses several prominent benchmarks for HAR. Notably, the Activity Net dataset, introduced in 2015, comprises 203 activity classes and an extensive 27,208 samples, achieving an impressive accuracy of 94.7% in recent evaluations [67,68]. The Kinetics-400 and Kinetics-700 datasets, from 2017 and 2019, respectively, include 400 and 700 classes with approximately 306,245 and 650,317 samples. These datasets are notable for their high accuracy rates of 92.1% and 85.9% [69,70,71]. The AVA dataset, also from 2017, contains 80 classes and 437 samples, with a recorded accuracy of 83.0% [72,73]. Datasets such as Kinetics and AVA are collected from YouTube videos. These datasets are affected by content availability issues due to reliance on external links, which may expire over time. The EPIC Kitchen 55 dataset from 2018 offers a comprehensive view with 149 classes and 39,596 samples. The Moments in Time dataset, released in 2019, is one of the largest, with 339 classes and around 1,000,000 samples, although it has a relatively lower accuracy of 51.2% [71,74]. Each dataset is instrumental for training and evaluating HAR models, providing diverse scenarios and activities.

**Table 2:** Benchmark datasets for HAR RGB and Skeleton.

Dataset	Data Set Modalities	Year	Class	Subject	Sample	Latest Accuracy
UPCV [75]	Skeleton	2014	10	20	400	99.20% [76]
Activity Net [67]	RGB	2015	203	-	27,208	94.7% [68]
Kinetics-400 [69]	RGB	2017	400	-	306,245	92.1% [71]
AVA [72]	RGB	2017	80	-	437	83.0% [73]
EPIC Kitchen 55 [77]	RGB	2018	149	32	39,596	-
AVE [78]	RGB	2018	28	-	4143	-
Moments in Times [74]	RGB	2019	339	-	1,000,000	51.2% [71]
Kinetics-700 [70]	RGB	2019	700	-	650,317	85.9% [71]
RareAct [79]	RGB	2020	122	905	2024	60.80% [80]
HiEve [81]	RGB, Skeleton	2020	-	-	-	95.5% [82]
MSRDaily Activity3D [83]	RGB, Skeleton	2012	16	10	320	97.50% [84]
N-UCLA [85]	RGB, Skeleton	2014	10	10	1475	99.10% [86]
Multi-View TJU [87]	RGB, Skeleton	2014	20	22	7040	-
UTD-MHAD [88]	RGB, Skeleton	2015	27	8	861	95.0% [89]
UWA3D Multiview II [90]	RGB, Skeleton	2015	30	10	1075	-
NTU RGB+D 60 [91]	RGB, Skeleton	2016	60	40	56,880	97.40% [86]
PKU-MMD [92]	RGB, Skeleton	2017	51	66	10,076	94.40% [93]
NEU-UB [94]	RGB	2017	6	20	600	-
Kinetics-600 [95]	RGB, Skeleton	2018	600	-	595,445	91.90% [71]
RGB-D Varing-View [96]	RGB, Skeleton	2018	40	118	25,600	-
NTU RGB+D 120 [97]	RGB, Skeleton	2019	120	106	114,480	95.60% [86]
Drive&Act [98]	RGB, Skeleton	2019	83	15	-	77.61% [99]
MMAct [100]	RGB, Skeleton	2019	37	20	36,764	98.60% [101]
Toyota-SH [102]	RGB, Skeleton	2019	31	18	16,115	-
IKEA ASM [103]	RGB, Skeleton	2020	33	48	16,764	-
ETRI-Activity3D [104]	RGB, Skeleton	2020	55	100	112,620	95.09% [105]
UAV-Human [106]	RGB, Skeleton	2021	155	119	27,428	55.00% [107]

### 3.2. Handcrafted Features with ML-Based Approach

Researchers employed handcrafted feature extraction with ML-based systems at an early age to develop HAR systems [108]. In the action representation step, the RGB data are utilized to transform into the feature vector, and these feature vectors are fed into the classifier [109,110] to obtain the desired results of the action classification step. Table 3 shows the analysis of the handcrafted-based approach, including the datasets, methods of feature extraction, classifier, years, and performance accuracy. Handcrafted features are designed to capture the physical motions performed by humans and the spatial and temporal variations depicted in videos that portray actions. These variations include methods that utilize the spatio-temporal volume-based representation of actions, methods based on Spatio-temporal Interest Points (STIPs), methods that rely on the trajectory of skeleton joints for action representation, and methods that utilize human image sequences for action representation. Chen et al. [111] demonstrate this by employing Depth Motion Map (DMM)-based gestures for motion information extraction, while Local Binary Pattern (LBP) feature encoding enhances discriminative power for action recognition. Meanwhile, Patel et al. [108] fuse various features, including Histogram of Oriented Gradients (HOG) and LBP, to improve network performance in recognizing human activities. The handcrafted features can be categorized as below:

#### 3.2.1. Holistic Feature Extraction

Holistic representation aims to capture the motion information of the entire human subject. Spatio-temporal action recognition often uses template-matching techniques, with key methods focusing on creating effective action templates. Bobick et al. introduced two approaches, Motion Energy Image (MEI) and Motion History Image (MHI), to perform action representation [126]. Meanwhile, Zhang et al. utilized polar coordinates in MHI and developed a Motion Context Descriptor (MCD) based on the Scale-Invariant Feature Transform (SIFT) [127]. Somasundaram et al. applied sparse representation and dictionary learning to calculate video self-similarity in both time and space [128]. In scenarios with a stationary camera, these approaches effectively capture shape-related information like human silhouettes and contours through background subtraction. However, accurately capturing silhouettes and contours in complex scenes or with camera movements remains challenging, especially when the human body is partially obscured. Many methods employ a sliding window approach to detect multiple actions within the same scene, which can be computationally expensive. These approaches transform dynamic human motion into a holistic representation in a single image. While they capture relevant foreground information, they are sensitive to background noise, including irrelevant information.

#### 3.2.2. Local and Global Representation

Holistic feature extraction techniques for HAR face several limitations, including sensitivity to background noise, reliance on stationary cameras, difficulty in complex scenes, occlusion issues, high computational cost, limited robustness to variations, and neglect of contextual information, making them less effective in dynamic, real-world scenarios.

Combining local and global representations can effectively address HAR’s holistic feature extraction limitations. Local features reduce background noise sensitivity and handle occlusions, while global features ensure comprehensive activity recognition. This combination enhances robustness to variations, manages complex scenes, and optimizes computational efficiency, improving HAR accuracy and reliability. The local presentation means identifying a specific region, while the global representation means identifying the whole region with significant motion information. These methods [14,15,16] contain local and global features based on spatial–temporal change trajectory attributes that are founded on key point tracking [17,129], motion changes that are derived from depth information [18,19], and action-based features that are predicated on human pose changes [20,21]. The HoG and HON4D [130] is one of the feature-based techniques that calculates features on the base orientation of gradients in an image or video sequence. The HoG features are then used to encode local and global texture information, aiming to recognize different actions. Some of the presented approaches exploit the HoG in action recognition, including [131,132,133,134], in various ways. HOF is a feature extraction method used in action recognition [135,136]. It involves building histograms to present different actions over the spatio-temporal domain in a video. However, in this method, the number of bins needs to be set in advance. The challenge addresses cluttered backgrounds and camera movement by performing a physical feature-driven approach to HOF.

#### 3.2.3. Classification Approach

Once we have the feature representation, we feed it into classifiers such as Support Vector Machine (SVM) [121], Random Forest, and K-Nearest Neighbor (KNN) [137,138] to predict the activity label. Some classification methods, such as Hidden Markov Models (HMMs), Condition Random Fields (CRFs) [139,140,141], Structured Support Vector Machine (SSVM) [110,142,143], and Global Gaussian Mixture Models (GGMMs) [7], perform sequentially for classification tasks. Additionally, Luo et al. utilized the features of fusion-based methods, Maximum Margin Distance Learning (MMDL) [144], and Multi-task Spare Learning Model (MTSLM) [145]. These methods perform the classification task based on combining various characteristics to enhance the classification task.

### 3.3. End-to-End Deep Learning Approach

The holistic, local, and global features reported promising results in the HAR task, but these handcrafted features need much specific knowledge to define relevant parameters. Additionally, they do not generalize sizable datasets well. In recent years, significant focus has been placed on utilizing DL in computer vision. Numerous approaches have used deep neural network-based methods to recognize human activity [22,23,47,146,147,148,149,150,151,152].

For example, Donahue et al. explored Long Short-Term Memory (LSTM) and developed Long-Term Recurrent Convolutional Networks (LRCNs) [153] to model CNN-generated spatial features across temporal sequences. Another significant HAR technique involves the use of LSTM with Convolution Neural Networks (CNNs) [154,155]. Ng et al. [155] introduced a Recurrent Neural Network (RNN) model to identify and classify the action, which performs a connection between the LSTM cell and the output of the underlying CNN. Donahue et al. [153] proposed a method of using long-term RNNs to map video frames of varying lengths to outputs of varying lengths, such as action descriptive text, rather than simply assigning them to a specific action category. Song et al. [156] introduced a model using RNNs with LSTM that employed multiple attention levels to discern key joints in the skeleton across each input frame.

Recently, researchers have utilized different ideas for spatio-temporal feature extraction, divided into four categories: two-stream networks, multi-stream networks, 3D CNN, and Hybrid Networks.

#### 3.3.1. Two Stream-Based Network

**Figure 6:**
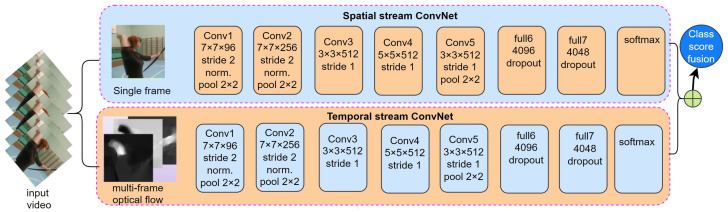
RGB-based two-stream architecture HAR [22].

The motion of an object can be represented based on the optical flow [157]. Simonyan et al. proposed a two-stream convolutional network to recognize human activity [22], as depicted in Figure 6. In a convolutional network with two streams, the optical flow information is computed from the sequence of images. Two separate CNNs process image and optical flow sequences as inputs during model training. Fusion of these inputs occurs at the final classification layer. The two-stream network handles a single-frame image and a stack of optical flow frames using 2D convolution. In contrast, a 3D convolutional network treats the video as a space–time structure and employs 3D convolution to capture human action features.

Numerous research endeavors have been conducted to enhance the efficacy of these two network architectures. Noteworthy advancements in the two-stream CNNs have been made by Zhang et al. [151], who substituted the optical flow sequence with the motion vector in the video stream. This substitution resulted in improved calculation speed and facilitated real-time implementation of the aforementioned HAR technique. The process of merging spatial and temporal information has been modified by Feichtenhofer et al. [50], shifting it from the initial final classification layer to an intermediate position within the network. As a result, the accuracy of action recognition has been further enhanced [51]. Moreover, an additional enhancement to the performance of the two-stream convolutional network was introduced through the proposal of a Temporal Segment Network (TSN). Moreover, the recognition results of TSN were further improved by the contributions of both Lan et al. [158] and Zhou et al. [159].

#### 3.3.2. Multi-Stream Based Network

RGB data paired with CNNs offers powerful action recognition capabilities. Liu et al. [160] leverage a multi-stream convolutional network to enhance recognition performance by incorporating manually crafted skeleton joint information with CNN-derived features. Shi et al. [53] employ transfer learning techniques in a three-stream network, incorporating dense trajectories to characterize long-term motion effectively. Attention mechanisms with RGB data focus on relevant regions for better action recognition. Shah et al. [66] propose a Generative Adversarial Network (GAN)-based knowledge distillation framework combining spatial attention-augmented EfficientNetB7 and multi-layer Gated Recurrent Units (GRUs) with handcrafted hybrid LBP features for robust HAR, while acknowledging limitations in model generalization and noise sensitivity in complex real-world environments.

#### 3.3.3. 3D CNN and Hybrid Networks

Traditional two-stream techniques often separate spatial and temporal information, which can render them less suitable for real-time deployment. These 3D approaches aim to address the limitations of the earlier two-stream networks. For example, Ji et al. [46] utilized the 3D CNN model for the action recognition task. This model extracts features from both the spatial and the temporal dimensions. Tran et al. [23] used C3D to extract spatio-temporal features for a large dataset to train the model, which is the extension of the 3DCNN model [46]. Carreira et al. [154] developed I3D, extending the network to extract spatio-temporal features along with the temporal dimension. They proposed image classification models to create 3D CNNs by transferring weights from 2D models pre-trained on ImageNet to align with the weights in the 3D model. P3D [161] and R(2+1)D [162] streamlined 3D network training using factorization, combining 2D spatial convolutions (1 × 3) with 1D temporal convolutions (3 × 1 × 1) instead of traditional 3D convolutions (3 × 3). For improved motion analysis, trajectory convolution [163] employed deformable convolutions in the temporal domain. Other approaches simplify 3D CNNs by integrating 2D and 3D convolutions within single networks to enhance feature maps, exemplified by models like MiCTNet [56], ARTNet [164], and S3D [165]. To enhance the performance of 3DCNN, CSN [166] has demonstrated the effectiveness of decomposing 3D convolution by separating channel interactions from spatio-temporal interactions, leading to state-of-the-art performance improvements. This technique can achieve speeds that are two to three times faster than previous methods. Feichtenhofer et al. developed the X3D method [167] that included both spatial and temporal dimensions with enhanced spatial, input resolution, and channel dimensions. Yang et al. [168] proposed that morphologically similar actions like walking, jogging, and running require discrimination assisted by visual speed. They proposed a Temporal Pyramid Network (TPN) similar to X3D. This approach enables the extraction of effective features at various temporal rates, reducing computational complexity while enhancing efficiency performances. Zhang et al. [169] proposed a 4D CNN with 4D convolution to capture the evolution of distant spatio-temporal representations.

Similarly, numerous researchers have made efforts to expand various 2D CNNs to 3D spatio-temporal structures to acquire knowledge about and identify human action features. Carreira et al. [154] expanded the network architecture of inception-V1 to incorporate 3D and introduced the two-stream inflated 3D ConvNet for HAR. Qin et al. [170] propose a fusion scheme combining classical descriptors with 3D CNN-learned features, achieving robustness against geometric and optical deformations. Diba et al. [171] extended DenseNet and introduced a temporal 3D ConvNet for HAR. Zhu et al. [172] expanded pooling operations across spatial and temporal dimensions, transforming the two-stream convolution network into a three-dimensional structure. Carreira et al. [154] conducted a comparison of five architectures: LSTM with CNN, 3D ConvNet, two-stream network, two-stream inflated 3D ConvNet, and 3D-fused two-stream network. In essence, 3D CNNs establish relationships between temporal and spatial features in various ways, complementing rather than replacing two-stream networks.

#### 3.3.4. Other Models

Hassan et al. [64] created a deep bidirectional LSTM model, which effectively integrates the advantages of temporal effective features extraction through Bi-LSTM and spatial feature extraction via CNN. Use of the LSTM architecture is not feasible for supporting parallel computing, which can limit its efficiency. To overcome this problem, the transformer architecture [173] has become popular in DL to address this limitation. Girdhar et al. [174] used the transformer-based architecture to add context features and developed an attention mechanism to improve performance. Khan et al. [65] present two end-to-end DL models—ConvLSTM and LRCN—for HAR from raw video inputs, leveraging time distributed layers for spatio-temporal encoding, while facing limitations in computational efficiency and real-time deployment on resource-constrained devices.

### 3.4. Mathematical Derivation of the Benchmark RGB-Based 3DCNN Method

In RGB-based HAR, each dataset sample can be represented as:

(1) $$D=\left\{\left(X_{1},\ldots ,X_{n}\right),\left(y_{1},\ldots ,y_{n}\right)\right\}$$

where $X_{i}$ is the *i*-th video example and $y_{i}$ is its corresponding ground truth label. Each video $X_{i}$ is typically represented as a tensor with dimensions (f,h,w,ch), where *f* is the number of frames per video, *h* and *w* denote the height and width of each frame, and ch is the number of color channels. CNNs are commonly used to process RGB data. They apply multiple convolutional layers to extract features from the input tensor. The feature at spatial location (i,j) in the *k*-th feature map at the *l*-th layer is calculated as [175]:

(2) $$z_{i,j,k}^{l}={\left(w_{k}^{l}\right)}^{T}x_{i,j}^{l}+b_{k}^{l}$$

where $w_{k}^{l}$ is the learned convolutional kernel for the *k*-th feature map at layer *l*, $x_{i,j}^{l}$ is the input patch at position (i,j) in layer *l*, $b_{k}^{l}$ is the bias term for the *k*-th feature map at layer *l*, and $z_{i,j,k}^{l}$ is the pre-activation value at position (i,j) in the *k*-th feature map. An activation function a(·) introduces non-linearity as flow:

(3) $$a_{i,j,k}^{l}=a\left(z_{i,j,k}^{l}\right)$$

where $a_{i,j,k}^{l}$ is the activated feature value at position (i,j) in the *k*-th feature map at layer *l* and a(·) is typically a ReLU, tanh, or sigmoid function. Pooling layers then reduce the resolution of the feature maps, enhancing shift-invariance and robustness as follows:

(4) $$y_{i,j,k}^{l}=pool\left(a_{m,n,k}^{l}\right),\;\forall \left(m,n\right)\in R_{ij}$$

where $y_{i,j,k}^{l}$ is the pooled feature at position (i,j) in the *k*-th feature map at layer *l*, $R_{ij}$ denotes the local pooling region around position (i,j), and pool(·) is typically a max or average pooling operation. After stacking several convolutional and pooling layers, fully connected layers are added to perform higher-level reasoning. The final output is often passed through a Softmax operator to produce class probabilities. The CNN model is trained by minimizing a task-specific loss function that measures the difference between predicted and true labels. Given *N* training samples $\left(x^{\left(n\right)},y^{\left(n\right)}\right)$, where n∈{1,2,…,N}, the overall loss function is given by [175]:

(5) $$L=\frac{1}{N}\sum_{n=1}^{N}\lambda \left(\theta ;y^{\left(n\right)},o^{\left(n\right)}\right)$$

where λ(·) denotes the chosen loss function (e.g., cross-entropy), θ represents all learnable model parameters (weights and biases), and $o^{\left(n\right)}$ is the model’s output prediction for the *n*-th sample. Training on the RGB modality can be expressed as:

(6) $$L\left(C\left(\phi_{m}\left(X_{i};\theta_{m}\right);\theta_{c}\right),y_{i}\right)$$

where $\phi_{m}\left(\cdot \right)$ is the CNN-based feature extractor for modality *m* with parameters $\theta_{m}$, C(·) is the classifier network with parameters $\theta_{c}$, L(·) denotes the loss function (e.g., cross-entropy), and $y_{i}$ is the ground truth label for the *i*-th sample.

#### 3.4.1. Three-Dimensional CNN

The 3D CNN processes spatio-temporal features by applying 3D convolutions across both spatial (height, width) and temporal (frames) dimensions. For a 3D convolution operation at a certain location (i,j,t) in the feature map [46] and also C3D [23] and P3D [161], we have:

(7) $$z_{i,j,t,k}^{l}=\sum_{m=-\delta_{h}}^{\delta_{h}}\sum_{n=-\delta_{w}}^{\delta_{w}}\sum_{p=-\delta_{t}}^{\delta_{t}}w_{k,m,n,p}^{l}\cdot x_{i+m,j+n,t+p}^{l-1}+b_{k}^{l}$$

where $w_{k,m,n,p}^{l}$ is the 3D convolutional kernel; $\delta_{h},\delta_{w},\delta_{t}$ are the spatial and temporal filter sizes; $x^{l-1}$ is the input feature map from the previous layer; and $b_{k}^{l}$ is the bias term.

#### 3.4.2. C3D [23]

C3D, also called a deep 3D ConvNet model, is similar to the VGG model [176], which is constructed with eight convolution layers, five max-pooling layers, and two fully connected layers, where all 3D convolution kernels are 3×3×3 and have a stride of 1 for both domains: spatial and temporal, whereas most pooling kernes are 2×2×2 and pooling 1 layers are 1×2×2. According to the experiment using the UCF101 test split-1 with different kernels for the temporal depth setting, 3D ConvNet achieves high accuracy, with 3×3×3 kernels, compared to the 2D ConvNet [23]. C3D achieved 85.2%, 85.2%, 78.3%, 98.1%, 87.7%, and 22.3% accuracy for the Sport1M, UCF101, ASLAN, YUPENN, UMD, and Object datasets, respectively [23].

#### 3.4.3. I3D (Inflated 3D ConvNet)

It is difficult to identify good video architectures due to the small video benchmarks (UCF101, HMDB51), and most methods obtain similar performance on this dataset. They then proposed the Kinetics Dataset and proposed I3D models. The I3D architecture followed the InceptionV1 (GoogleNet) architecture. I3D inflates 2D filters from a pre-trained 2D CNN (e.g., from ImageNet) into 3D convolutions to capture temporal relationships. For the convolution at position (i,j,t), the equation is:

(8) $$z_{i,j,t,k}^{l}={\left(w_{k}^{l}\right)}^{T}\cdot x_{i,j,t}^{l-1}+b_{k}^{l}$$

where $w_{k}^{l}$ is the inflated 3D kernel and $x_{i,j,t}^{l-1}$ represents the input features across the spatial and temporal dimensions. The model design is based on Inception V1, so that when training I3D, the I3D weights can be initialized with the InceptionV1 weights, which are pretrained in ImageNet [154].

#### 3.4.4. S3D

The S3D model was developed to overcome the computational challenges of I3D. While I3D delivers strong performance, it is very computationally expensive. This leads to some important questions: Is 3D convolution truly necessary? If so, which layers should use 3D convolution, and which could use 2D convolution instead? These choices might depend on the nature of the dataset and the specific task. Additionally, is it crucial to apply convolution over both time and space together, or would it be enough to handle these dimensions separately? To address these issues, Xie et al. introduced the S3D model [165]. This model replaces traditional 3D convolutions with spatial and temporal separable convolutions, helping reduce computational costs while still effectively capturing both spatial and temporal features in video data. The convolution at position (i,j,t) in S3D is given by the following equation:

(9) $$z_{i,j,t,k}^{l}=\sum_{m=-\delta_{h}}^{\delta_{h}}\sum_{n=-\delta_{w}}^{\delta_{w}}\sum_{p=-\delta_{t}}^{\delta_{t}}w_{k,m,n,p}^{l}\cdot x_{i+m,j+n,t+p}^{l-1}+b_{k}^{l}$$

where $w_{k,m,n,p}^{l}$ represents the 3D kernel and $x^{l-1}$ is the input feature map from the previous layer. This method improves efficiency, accuracy, and speed by reducing unnecessary computation.

#### 3.4.5. R3D, R(2+1)D

Despite ResNet152 having only a 2D convolutional layer, it outperforms C3D on the video dataset. The development of a very deep 3D CNN from scratch results in expensive computational cost and memory demand. Three-dimensional convolution vs. (2+1)D convolution, which one is better? It is difficult to say, but we can explain as follows. Full 3D convolution used filter size t×d×d, where d represents the height and width of the RGB image and *t* represents the temporal extent. On the other hand, (2+1)D splits the total operation into two sections, 2D convolution and 1D convolution, to reduce the computational complexity. R(2+1)D [162] uses a factorized 3D convolution: 2D convolutions in the spatial domain and 1D convolutions in the temporal domain. The update rule is:

(10) $$z_{i,j,t,k}^{l}=\sum_{m=-\delta_{h}}^{\delta_{h}}\sum_{n=-\delta_{w}}^{\delta_{w}}w_{\mathrm{spatial}}\cdot x_{i+m,j+n,t}^{l-1}+\sum_{p=-\delta_{t}}^{\delta_{t}}w_{\mathrm{temporal}}\cdot x_{i,j,t+p}^{l-1}+b_{k}^{l}$$

where $w_{\mathrm{spatial}}$ is the 2D spatial kernel and $w_{\mathrm{temporal}}$ is the 1D temporal kernel.

#### 3.4.6. P3D ResNet

Qiu et al. [161] proposed a novel architectural design termed Pseudo-3D ResNet (P3D ResNet), wherein each block is assembled in a distinct ResNet configuration. P3D is a pseudo 3D Convolutional Neural Network (3DCNN) constructed by combining C3D and ResNet architectures. For the Sports-1M dataset, P3D-ResNet achieves a performance accuracy of 66.4%, outperforming individual ResNet and C3D models, which achieve accuracies of 64.6% and 61.1%, respectively [161]. P3D can be categorized into three versions: P3D-A, which feeds spatial features into the temporal module and then concatenates with the residual unit. P3D-B is constructed by parallel concatenation of spatial, temporal, and residual units. P3D-C, which concatenates spatial, spatial–temporal, and residual units.

#### 3.4.7. SlowFast Networks

SlowFast Networks use two streams, where a slow stream processes fewer frames per second (capturing long-term dependencies) and a fast stream processes more frames per second (capturing fast motion). The model was proposed by the Facebook AI Research Team and published in 2019 [177]. This model is inspired by biological studies on retinal ganglion cells. The outputs from both streams are fused. This network addresses low frame rate issues, where the slow pathway has low temporal resolution, and the fast pathway has high frame rate and α× temporal resolution. However, the fast pathway remains lightweight in terms of computational complexity due to the use of β or $\frac{1}{8}$ of the channels, and it fuses the latest connections. The equations for the combined feature map are:

(11) $$z_{i}^{\mathrm{slow}}=\sum_{m=-\delta_{h}}^{\delta_{h}}\sum_{n=-\delta_{w}}^{\delta_{w}}w_{\mathrm{slow}}\cdot x_{i}+b_{\mathrm{slow}}$$

(12) $$z_{i}^{\mathrm{fast}}=\sum_{m=-\delta_{h}}^{\delta_{h}}\sum_{n=-\delta_{w}}^{\delta_{w}}w_{\mathrm{fast}}\cdot x_{i}+b_{\mathrm{fast}}$$

where $w_{\mathrm{slow}}$ and $w_{\mathrm{fast}}$ are the weights for slow and fast streams and $x_{i}$ is the input feature map. The slow and fast streams are then fused together to generate the final output. The Slow pathway uses a large temporal stride on input frames to focus on spatial information. It captures fine details and spatial patterns, similar to the parvocellular (P cell) function in retinal ganglion cells. This helps understand long-term data structure. The Fast pathway, with a smaller temporal stride, focuses on capturing temporal information. It has lower channel capacity compared to the Slow pathway and resembles the magnocellular (M cell) function in retinal ganglion cells. It specializes in detecting rapid visual changes, making it ideal for processing fast motion. What sets the Fast pathway apart is its ability to maintain good accuracy with a much lower channel capacity, making it efficient and lightweight in the SlowFast model.

#### 3.4.8. X3D

This model was also proposed by Facebook AI Research and published in 2020 [167]. The basic network architecture is designed according to the ResNet and Fast pathway design for SlowFast networks. They have six expansion factors: $\gamma_{\tau }$, $\gamma_{t}$, $\gamma_{s}$, $\gamma_{w}$, $\gamma_{b}$, and $\gamma_{d}$. X-Fast increases the temporal activation size, $\gamma_{t}$, and frame-rate, $1/\gamma_{\tau }$, to improve temporal resolution while keeping the clip duration constant. X-Temporal extends both the duration and temporal resolution by sampling longer clips and increasing the frame-rate, $1/\gamma_{\tau }$. X-Spatial enhances the spatial resolution, $\gamma_{s}$, by improving the spatial sampling resolution of the input video. X-Depth increases the network’s depth by adding more layers per residual stage by $\gamma_{d}$. X-Width expands the number of channels uniformly across all layers using a global width factor, $\gamma_{w}$. Lastly, X-Bottleneck increases the inner channel width, $\gamma_{b}$, of the center convolutional filter in each residual block to improve processing efficiency. This paper presents X3D, a family of efficient video networks that progressively expand a tiny 2D image classification architecture along multiple network axes, in space, time, width, and depth. X3D [167] is a scalable architecture for video recognition. It adjusts the depth, width, and resolution of the 3D convolutions based on the computational resources. To expand the Basis Network to X3D, the model’s complexity, *C*, is first determined, where $C_{base}$ represents the initial complexity and ΔC denotes the desired increase. Each parameter $p_{i}$ (for i=1,2,…,6) is incrementally expanded until the model reaches the target complexity $C_{target}$, defined as:

(13) $$C_{target}=C_{base}+\Delta C$$

Six models, $M_{i}$, are created, corresponding to each expanded parameter. The models are trained, and their performance is evaluated using a performance metric $P(M_{i})$. The best-performing model, $M^{*}$, is selected:

(14) $$M^{*}=\arg\max_{M_{i}}P\left(M_{i}\right)$$

This best model, $M^{*}$, becomes the new basis network, and the process can be repeated if further refinement is needed [167].

#### 3.4.9. Vision Transformer (ViT)

Vision Transformer (ViT) is one of the crucial models designed for image classification tasks, utilizing a Transformer architecture originally developed for Natural Language Processing (NLP) [178]. ViT processes images by dividing them into non-overlapping patches and treating these patches as input tokens for a Transformer model, similar to how words are treated in text. Given that we are processing a batch of 100 videos, each with 32 frames, the input tensor *V* will have the shape:

(15) $$V\in R^{100\times 32\times H\times W\times C}$$

where *H* and *W* are the height and width of each frame and *C* is the number of channels (e.g., 3 for RGB). For each video, *V*, we will extract patches from each frame. For each frame $I_{t}\in R^{H\times W\times C}$, we need to divide it into *N* non-overlapping patches, and we embed them into a patch embedding space:

(16) $$P_{t}={\left\{p_{t,i}\right\}}_{i=1}^{N}$$

Each patch $p_{t,i}$ is linearly projected to a fixed-dimensional vector $e_{t,i}$ using a convolutional layer:

(17) $$e_{t,i}=\mathrm{Embed}\left(p_{t,i}\right)$$

Now, for each frame *t*, the patch embeddings will result in:

(18) $$E_{t}=\left[e_{t,1},e_{t,2},\ldots ,e_{t,N}\right]$$

The full video sequence *V* now becomes a sequence of spatial tokens, with patches from all 32 frames:

(19) $$X=[E_{1};E_{2};\ldots ;E_{32}]$$

Thus, the shape of the final tokenized video sequence will be:

(20) $$X\in R^{100\times 32N\times D}$$

where *D* is the dimensionality of each patch embedding. Then, multi-head self-attention is applied to the tokenized sequence of video frames. The attention mechanism computes the output sequence of tokens by considering the relationships between the tokens across both time and space:

(21) $$\mathrm{Attention}\left(X\right)=\mathrm{Softmax}\left(\frac{QK^{T}}{\sqrt{d_{k}}}\right)V$$

where Q,K,V are the query, key, and value matrices, and $d_{k}$ is the dimension of the key vectors. After the self-attention operation, the model aggregates the output tokens (using global pooling, class token, or other aggregation methods) and projects it into the final class prediction:

(22) $$\hat{y}=\mathrm{Softmax}\left(W\cdot z\right)$$

where *z* is the final token representation after the Transformer encoder layers and *W* is the classification weight matrix.

#### 3.4.10. Video Vision Transformer (ViViT)

ViViT [179] adapts the Vision Transformer (ViT) architecture for video data by extracting *N* non-overlapping spatio-temporal “tubes” from the input video, denoted as $x_{1},x_{2},\ldots ,x_{N}\in R^{t\times h\times w\times c}$. Here, *N* is determined by the following expression:

(23) $$N=\left\lfloor \frac{T}{t}\right\rfloor \times \left\lfloor \frac{H}{h}\right\rfloor \times \left\lfloor \frac{W}{w}\right\rfloor ,$$

where *T*, *H*, and *W* represent the temporal and spatial dimensions (time, height, and width) of the video and *t*, *h*, and *w* are the corresponding dimensions of each tube.

Each tube $x_{i}$ is then transformed into a token $z_{i}\in R^{d}$ by a linear mapping operator *E*, as follows:

(24) $$z_{i}=Ex_{i}.$$

These tokens are concatenated together to form a sequence. A learnable class token, $z_{\mathrm{cls}}\in R^{d}$, is prepended to this sequence. Since transformers are permutation-invariant, a positional embedding $p\in R^{(N+1)\times d}$ is added to the sequence. Consequently, the tokenization process can be represented as:

(25) $$z^{0}=\left[z_{\mathrm{cls}},Ex_{1},Ex_{2},\ldots ,Ex_{N}\right]+p.$$

Here, $z^{0}$ represent the token where *z* can be the sequence of the tokents, $(z_{\mathrm{cls}}$ represent the learnable class token, *x* is the input data with ith position and *E* represent the mapping operator, and p represent the positional embedding. It is important to note that the linear projection *E* can be interpreted as a 3D convolution with a kernel size of t×h×w and a stride of (t,h,w) in the time, height, and width dimensions, respectively.

The resulting sequence of tokens, *z*, is then passed through a transformer encoder consisting of *L* layers. At each layer *ℓ*, the following operations are applied sequentially:

(26) $$y^{l}=\mathrm{MSA}\left(\mathrm{LN}\left(z^{l-1}\right)\right)+z^{l-1},$$

(27) $$z^{l}=\mathrm{MLP}\left(\mathrm{LN}\left(y^{l}\right)\right)+y^{l}$$

where MSA represents multi-head self-attention [173], LN refers to layer normalization, and MLP consists of two linear layers with a GeLU non-linearity in between.

Finally, a linear classifier, $W^{\mathrm{out}}\in R^{d\times C}$, maps the output of the encoded classification token, $z_{\mathrm{cls}}^{L}$, to one of *C* possible classes.

The experimental results demonstrate the effectiveness of ViViT, with the ViViT-B model backbone and a tubelet size of 16 × 216 × 2 achieving high performance. The model is evaluated on two well-known benchmarks: Top-1 accuracy on Kinetics 400 (K400) and action accuracy on Epic Kitchens (EK). The runtime is measured during inference on a TPU-v3, showcasing the model’s efficiency and scalability [179].

#### 3.4.11. Multiview Transformers for Video Recognition (MVT)

MVT [180] introduces a novel approach to video recognition by applying transformers to multiple views of the same video. By leveraging information from different perspectives, MVT improves recognition performance and robustness across various viewpoints. This approach enhances the model’s ability to capture diverse spatial and temporal patterns, making it particularly effective in complex video recognition tasks. MVT combines the strengths of two well-established models: SlowFast and ViViT. The SlowFast model, known for its ability to handle both fast and slow motions in video data, is integrated with ViViT, a transformer-based model that efficiently captures spatial and temporal dependencies in video. The MVT for video recognition based on the scenario in Equation (15) extracts multiple sets of tokens, $z^{0,(1)},z^{0,(2)},\ldots ,z^{0,(V)}$, from the input video, where *V* is the number of views. Each view’s tokens are processed independently by separate transformer encoders with lateral connections for cross-view fusion. The tokens from all views are concatenated to form a unified sequence:

(28) $$Z^{0}=\left[z_{\left(1\right)}^{0},z_{\left(2\right)}^{0},\ldots ,z_{\left(V\right)}^{0}\right]$$

Due to the prohibitive computational cost of performing self-attention on all tokens from all views, a more efficient method is introduced by fusing tokens between adjacent views using Cross-View Attention (CVA). This is performed by applying attention where queries come from the larger view and keys and values from the smaller view, after projecting them to the same dimension [180]:

(29) $$z^{\left(i\right)}=\mathrm{CVA}\left(z^{\left(i\right)},W_{\mathrm{proj}}z^{\left(i+1\right)}\right)$$

The attention is computed as [180]:

(30) $$\mathrm{CVA}\left(x,y\right)=\mathrm{Softmax}\left(\frac{W^{Q}x{\left(W^{K}y\right)}^{⊤}}{\sqrt{d_{k}}}\right)W^{V}y$$

#### 3.4.12. UniFormer-Based 3DCNN

UniFormer [181] is a new model that combines the advantages of CNNs and Vision Transformers (ViTs) to solve the challenges of learning representations from images and videos. CNNs reduce local redundancy but struggle to capture global dependencies due to their limited receptive fields. ViTs, on the other hand, capture long-range dependencies but suffer from high redundancy from comparing all tokens. UniFormer solves this by introducing a unique block structure with local and global token affinity, addressing both redundancy and dependency. A UniFormer block consists of three modules: Dynamic Position Embedding (DPE), Multi-Head Relation Aggregator (MHRA), and Feed Forward Network (FFN). This design allows UniFormer to handle both image and video inputs efficiently. UniFormer is highly versatile, working well for various vision tasks, from image classification to video understanding. It achieves state-of-the-art results across several benchmarks and outperforms traditional models like 3D CNNs without needing extra training data. UniFormer achieves 86.3% top-1 accuracy on ImageNet-1K classification and demonstrates state-of-the-art performance in various tasks, including 82.9%/84.8% top-1 accuracy on Kinetics-400/600, 60.9%/71.2% top-1 accuracy on Sth-Sth V1/V2, 53.8 box AP and 46.4 mask AP on COCO object detection, 50.8 mIoU on ADE20K semantic segmentation, and 77.4 AP on COCO pose estimation.

#### 3.4.13. VideoMAE Based 3DCNN

VideoMAE (Video Masked Autoencoders) [182] is a self-supervised pre-training method designed to learn video representations efficiently, particularly on small datasets. Inspired by ImageMAE, VideoMAE uses a high masking ratio (90–95%) for video tube masking, which makes the reconstruction task more challenging. This higher masking ratio is possible due to the temporally redundant nature of videos, allowing VideoMAE to learn effective representations by focusing on the most informative content. VideoMAE shows that it can achieve impressive performance with a small number of training videos (around 3–4 k) without relying on additional data, unlike traditional models that require large-scale datasets. The model also demonstrates that the quality of data is more important than the quantity when it comes to self-supervised video pre-training. With this approach, VideoMAE achieves remarkable results on benchmarks like Kinetics-400 (87.4%), Something-Something V2 (75.4%), and UCF101 (91.3%) without any extra data.

#### 3.4.14. InternVideo 3DCNN Model

InternVideo [183] is a general video foundation model that combines generative and discriminative self-supervised learning to improve video understanding. It utilizes two main pretraining objectives: masked video modeling and video-language contrastive learning. By selectively coordinating these complementary frameworks, InternVideo efficiently learns video representations that boost performance in various video applications. This approach allows InternVideo to achieve state-of-the-art results across 39 video datasets, covering tasks like action recognition, video-language alignment, and open-world video tasks. Notably, it achieves impressive top-1 accuracies of 91.1% on Kinetics-400 and 77.2% on Something-Something V2, demonstrating its effectiveness and general applicability for video understanding.

## 4. Skeleton Data Modality-Based Action Recognition Method

The main challenges of the RGB-based data modality-based HAR system are redundant background and computational complexity issues, and the skeleton-based data modality helps us overcome these challenges. In addition, coupling with joint coordinate estimation algorithms such as OpenPose and SDK [184] has improved the performance of accuracy and reliability of the skeleton data. Skeleton data obtained from the joint position offer several benefits over the RGB data, such as illumination variations, viewing angles, and background occlusions, making it less susceptible to noise interference. The research prefers to perform HAR by using skeleton data because they provide more focused information and reduce redundancy. Based on the feature extraction methods for HAR, the skeleton data can be divided into DL-based methods, relying on learned features, and ML-based methods, which use handcrafted features. In addition, the skeleton data depend on the precise joint position and pose estimation techniques.

**Figure 7:**
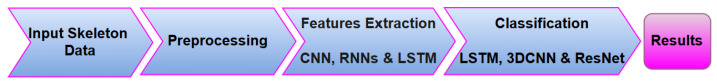
Workflow of skeleton-based action recognition.

Figure 7 shows the framework of skeleton-based approaches. Table 4 describes key information regarding the skeleton-based data modality on the existing model, including datasets, classification methods, years, and performance accuracy. We describe the well-known pose estimation algorithms in the following section.

### 4.1. Skeleton-Based HAR Dataset

We provided the most popular benchmark HAR datasets, which come from the skeleton, which is demonstrated in Table 2. The dataset table demonstrates details of the datasets, including modalities, creation year, number of classes, number of subjects who participated in recording the dataset, number of samples, and the latest performance accuracy of the dataset with citations. The Skeleton dataset includes a variety of notable benchmarks essential for HAR. The UPCV dataset from 2014 features 10 classes, 20 subjects, and 400 samples, achieving an outstanding accuracy of 99.2% [75,76]. The NTU RGB+D dataset, introduced in 2016 and expanded in 2019, is one of the most comprehensive, with 60 and 120 classes, 40 and 106 subjects, and 56,880 and 114,480 samples, respectively, both versions recording an accuracy of 97.4% [86,91,97]. The MSRDailyActivity3D dataset from 2012 includes 16 classes, 10 subjects, and 320 samples, with an accuracy of 97.5% [83,84]. The PKU-MMD dataset from 2017 contains 51 classes, 66 subjects, and 10,076 samples, with a notable accuracy of 94.4% [92,93]. The Multi-View TJU dataset from 2014 offers 20 classes, 22 subjects, and 7040 samples. These datasets are crucial for training and testing HAR models, offering diverse activities and scenarios to enhance model robustness and accuracy.

### 4.2. Pose Estimation

**Figure 8:**
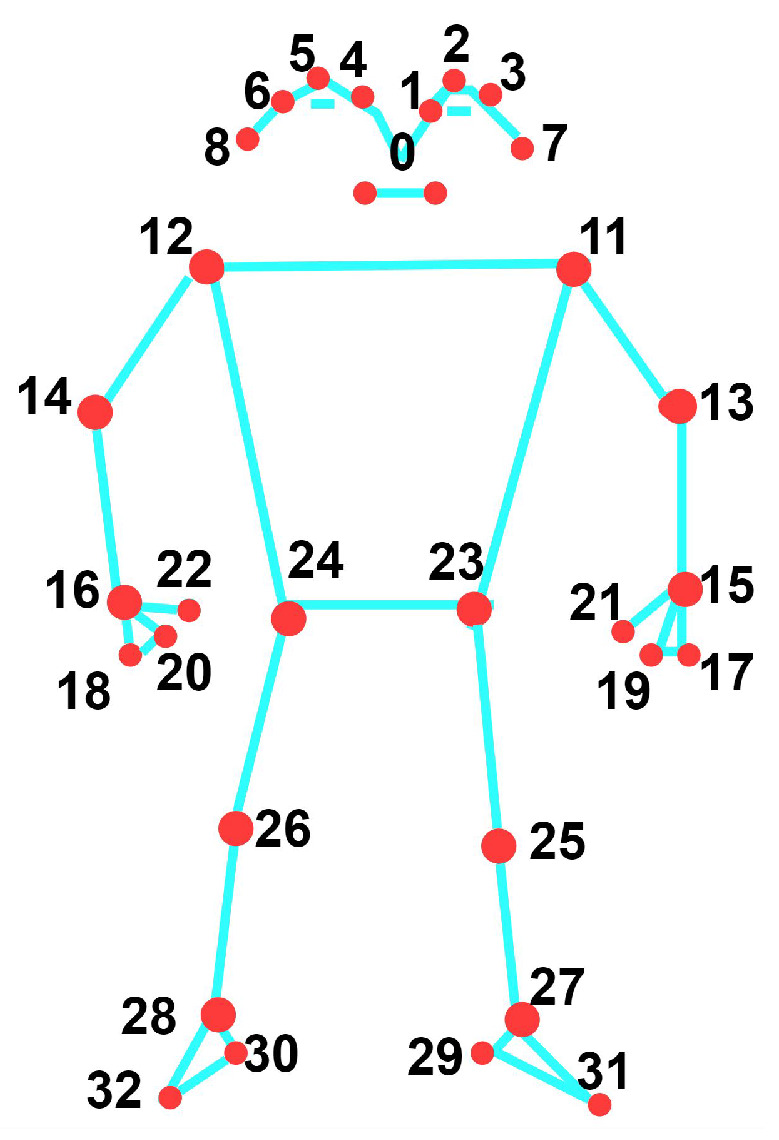
Positions of the 33 key landmarks on the human body. (0) Nose, (1) Left eye inner, (2) Left eye, (3) Left eye outer, (4) Right eye inner, (5) Right eye, (6) Right eye outer, (7) Left ear, (8) Right ear, (9) Mouth left, (10) Mouth right, (11) Left shoulder, (12) Right shoulder, (13) Left elbow, (14) Right elbow, (15) Left wrist, (16) Right wrist, (17) Left pinky, (18) Right pinky, (19) Left index, (20) Right index, (21) Left thumb, (22) Right thumb, (23) Left hip, (24) Right hip, (25) Left knee, (26) Right knee, (27) Left ankle, (28) Right ankle, (29) Left heel, (30) Right heel, (31) Left foot index, (32) Right foot index.

We can extract human joint skeleton points from the RGB video using media pipe, openpose, AlphaPose [185,186], MMPose, etc. Using a media pipe, Figure 8 demonstrates the 33 joint skeleton points for the whole body. Human limb trunk reconstruction includes estimating human pose by detecting joint positions in the skeleton and establishing their connections. Traditional methods, relying on manual feature labeling and regression for joint coordinate retrieval, suffer from low accuracy and are highly sensitive to occlusion. DL-based methods, including 2D and 3D pose estimation, have become pivotal in this research domain.

#### 4.2.1. Two-Dimensional Human Pose Estimation-Based Methods

The objective of 2D human pose estimation is to identify significant body parts in an image and connect them sequentially to form a human skeleton graph. Research commonly addresses the classification of single and multiple human subjects. In single-person pose estimation, the goal is to detect a solitary individual in an image. This involves first recognizing all joints of the person’s body and subsequently generating a bounding box around them. Two main categories of models exist for single-person pose estimation. The first utilizes a direct regression approach, where key points are directly predicted from extracted features. In 2D pose estimation, one can employ deformable part models to recognize the object by matching a set of templates. Nevertheless, these deformable part models exhibit limited expressiveness and fail to consider the global context. Yan et al. [187] proposed a pose-based approach and performed two main methods: detection-based and regression-based approaches. Detection-based methods utilize powerful part detectors based on CNNs, which can be integrated using graphical models, as described by Yuille et al. [188]. For solving the detection problem, pose estimation can be represented as a heat map where each pixel indicates the detection confidence of a joint, as outlined by Bulat et al. [189]. However, detection approaches do not directly provide joint coordinates. A post-processing step is applied to recover poses where (x, y) coordinates are obtained by utilizing the max function. Toshev et al. [190] proposed a cascade of regressor methods to estimate poses; they employ the regression-based approach with a nonlinear function that maps the joint coordinates and refines pose estimates. Carreira et al. [191] proposed the Iterative Error Feedback (IEF) approach, where iterative prediction is performed to correct the current estimates. Instead of predicting outputs in a single step, a self-correcting model is employed, which modifies an initial solution by incorporating error predictions, also called IEF. However, the sub-optimal nature of the regression function leads to lower performance than detection-based techniques.

#### 4.2.2. Three-Dimensional Human Pose Estimation-Based Methods

Conversely, when presented with an image containing an individual, the objective of 3D pose estimation is to generate a 3D pose that accurately aligns with the spatial location of the person depicted. The accurate reconstruction of 3D poses from real-life images holds significant potential in various fields of HAR, such as entertainment and human–computer interaction, particularly indoors and outdoors. Earlier approaches relied on feature engineering techniques, whereas the most advanced techniques are based on deep neural networks, as proposed by Zhou et al. [192] Three-dimensional pose estimation is acknowledged to be more complex than its 2D handle due to its management of a larger 3D pose space and an increased number of ambiguities. Nunes et al. [193] presented skeleton extraction through depth images, wherein skeleton joints are inferred frame by frame. A manually selected set of 15 skeleton joints, as determined by Gan et al. [112], are used to form an APJ3D representation, which is based on relative positions and local spherical angles. These 15 joints, which have been deliberately selected, play a crucial role in the development of a concise representation of human posture. Spatial features are encoded using diverse metrics, including joint distances, orientations, vectors, distances between joints and lines, and angles between lines. These measures collectively contribute to a comprehensive texture feature set, as suggested by Chen et al. [194]. Additionally, a CNN-based network is trained to recognize corresponding actions.

**Table 4:** Skeleton-based action recognition methods using handcrafted and deep learning approaches.

Author	Year	Dataset Name	Modality	Method	Classifier	Accuracy [%]
Veeriah et al. [195]	2015	MSRAction3D KTH-1 (CV) KTH-2 (CV)	Skeleton	Differential RNN	Softmax	92.03 93.96, 92.12
Xu et al. [116]	2016	MSRAction3D UTKinect Florence3D action	Skeleton	SVM with PSO	SVM	93.75 97.45, 91.20
Zhu et al. [196]	2016	SBU Kinect HDM05, CMU	Skeleton	Stacked LSTM	Softmax	90.41 97.25, 81.04
Li et al. [197]	2017	UTD-MHAD NTU-RGBD (CV) NTU-RGBD (CS)	Skeleton	CNN	Maximum Score	88.10 82.3 76.2
Soo et al. [198]	2017	NTU-RGBD (CV) NTU-RGBD (CS)	Skeleton	Temporal CNN	Softmax	83.1 74.3
Liu et al. [160]	2017	NTU-RGBD (CS) NTU-RGBD (CV) MSRC-12 (CS) Northwestern-UCLA	Skeleton	Multi-stream CNN	Softmax	80.03, 87.21 96.62, 92.61
Das et al. [199]	2018	MSRDailyActivity3D NTU-RGBD (CS) CAD-60	Skeleton	Stacked LSTM	Softmax	91.56 64.49, 67.64
Si et al. [200]	2019	NTU-RGBD (CS) NTU-RGBD (CV) UCLA	Skeleton	AGCN-LSTM	Sigmoid	89.2, 95.0 93.3
Shi et al. [201]	2019	NTU-RGBD (CS) NTU-RGBD (CV) Kinetics	Skeleton	AGCN	Softmax	88.5 95.1 58.7
Trelinski et al. [202]	2019	UTD-MHAD MSR-Action3D	Skeleton	CNN-based	Softmax	95.8, 77.44 80.36
Li et al. [203]	2019	NTU-RGBD (CS) Kinetics (CV)	Skeleton	Actional graph based CNN	Softmax	86.8 56.5
Huynh et al. [204]	2019	MSRAction3D UTKinect-3D SBU-Kinect Interaction	Skeleton	ConvNets	Softmax	97.9 98.5, 96.2
Huynh et al. [205]	2020	NTU-RGB+D UTKinect-Action3D	Skeleton	PoT2I with CNN	Softmax	83.85, 98.5
Naveenkumar et al. [206]	2020	UTKinect-Action3D NTU-RGB+D	Skeleton	Deep ensemble	Softmax	98.9, 84.2
Plizzari et al. [207]	2021	NTU-RGBD 60 NTU-RGBD 120 Kinetics Skeleton-400	Skeleton	ST-GCN	Softmax	96.3, 87.1 60.5
Snoun et al. [208]	2021	RGBD-HuDact, KTH	Skeleton	VGG16	Softmax	95.7, 93.5
Duan et al. [209]	2022	NTU-RGBD UCF101	Skeleton	PYSKL	-	97.4, 86.9
Song et al. [210]	2022	NTU-RGBD	Skeleton	GCN	Softmax	96.1
Zhu et al. [211]	2023	UESTC NTU-60 (CS)	Skeleton	RSA-Net	Softmax	93.9, 91.8
Zhang et al. [212]	2023	NTU-RGBD Kinetics-Skeleton	Skeleton	Multilayer LSTM	Softmax	83.3 27.8 (Top-1) 50.2 (Top-5)
Liu et al. [213]	2023	NTU-RGBD 60 (CV)NTU-RGBD 120 (CS)	Skeleton	LKJ-GSN	Softmax	96.1 86.3
Liang et al. [214]	2024	NTU-RGBD (CV) NTU-RGBD 120 (CS) FineGYM	Skeleton	MTCF	Softmax	96.9, 86.6 94.1
Karthika et al. [215]	2025	NTU-RGBD 60 NTU-RGBD 120 Kinetics-700 Micro- Action-52	Skeleton	Stacked Ensemble	Logistic Regression	97.87 98.0 97.50 95.20
Sun et al. [216]	2025	Self collected KTH UTD-MHAD	Skeleton	Multi channel fussion	Logistic Regression	98.16 92.85 84.98
Mehmood et al. [217]	2025	NTU-RGB+D (CS/CV) Kinetics UCF-101 HMDB-51	Skeleton	EMS-TAGCN	Logistic Regression	91.3/97.5 62.3 51.24 72.7

### 4.3. Handcrafted Feature and ML-Based Classification Approach

Researchers determine handcrafted features using statistical features extracted from action data. These features describe the dynamics or statistical properties of the action analyzed. Yang et al. [18] proposed a method to extract the super vector features to determine the action based on the depth information. Shao et al. [218] combined shape and motion information for HAR through temporal segmentation, utilizing MHI and Predicted Gradients (PCOG) as feature descriptors. Yang et al. [219] introduced the Depth Motion Map (DMM) technique, which allows for the projection and compression of the spatio-temporal depth structure from different viewpoints, including the side, front, and upper views. This process results in the formation of three distinct motion history maps. To represent these motion history maps, the authors employed the HOG feature. Instead of using HOG, Chen et al. [111] employed local features to describe human activities based on Dynamic Motion Models (DMMs). Additionally, Chen et al. [220] introduced a spatio-temporal depth layout across frontal, lateral, and upper orientations. Departing from depth compression methods, they extracted motion trajectory shapes and boundary histogram features from spatio-temporal interest points, leveraging dense sampling and joint points in each perspective to depict actions. Moreover, Miao et al. [221] applied the discrete cosine variation technique for the effective compression of depth maps. Simultaneously, they generated action features by utilizing transform coefficients. From the available depth data, it is possible to estimate the structure of the human skeleton promptly and precisely. Shotton et al. [222] proposed a method for the real-time estimation of body postures from depth images, thereby facilitating the rapid segmentation of humans based on depth. Within this context, the problem of detecting joints has been simplified to a per-pixel classification task. Additionally, there is ongoing research in the field of HAR that employs depth data and focuses on methods utilizing the human skeleton. These approaches analyze changes in the joint points of the human body across consecutive video frames to characterize actions, encompassing alterations in both the position and appearance of the joint points. Xia et al. [223] proposed a three-dimensional joint point histogram as a means to depict the human pose and subsequently formulated the action using a discrete hidden Markov model. Keceli et al. [224] captured depth and human skeleton information via employment of the Kinect sensor, and subsequently derived human action features by assessing angle and displacement information regarding the skeleton joint points. Similarly, Yang et al. [21] developed a method based on the EigenJoints, which leverages an Accumulative Motion Energy (AME) function to identify video frames and joint points that offer richer information for action modeling. Pazhoumand et al. [225] utilized the longest common subsequence method to select distinctive features with high discriminatory power from the skeleton’s relative motion trajectories, thereby providing a comprehensive description of the corresponding action.

Handcrafted features offer high interpretability and simplicity and are straight-forward to use. However, the handcrafted feature-based method requires prior knowledge, which is difficult to generalize.

### 4.4. End-to-End Deep Learning-Based Approach

**Figure 9:**
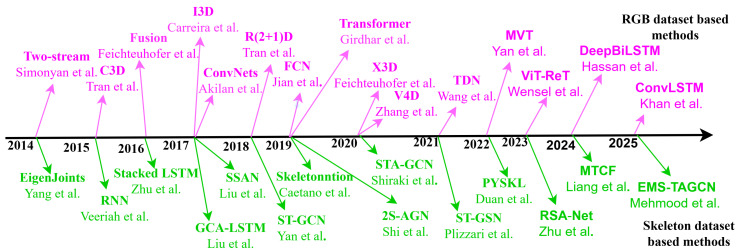
Milestone approaches for HAR: RGB-based milestone methods are in pink font [22,23,50,52,57,61,63,64,65,154,162,167,169,174,180] while skeleton-based milestone methods are in green font [21,160,195,196,201,207,209,211,214,217,228,233,244,245].

Recently, there has been a growing acknowledgment in HAR of the advantages of integrating skeleton data with DL-based techniques. The handcrafted features have reduced discriminative capability for HAR; conversely, to extract features efficiently, the utilization of methods based on DL necessitates a substantial quantity of training data. Figure 9 demonstrates the year-wise end-to-end DL-based method developed by various researchers for RGB and skeleton-based HAR systems. As shown, several notable models leveraging Recurrent Neural Networks (RNNs), CNN, and GCN have been developed.

#### 4.4.1. CNN-Based Methods

Skeleton data combined with ML methods provide efficient action recognition capabilities. Zhang et al. [226] utilized the Kinect sensor to capture skeletal representations, enabling the recognition of actions based on body part movements. Skeleton data paired with CNNs offer robust action recognition. As a result, in the work of Wang et al. [47], an advantage is found in combining handcrafted and DL-based features through the use of an enhanced trajectory. Additionally, the Trajectory-pooled Deep-Convolutional Descriptor (TpDD), also referred to as Two-stream ConvNets, is employed. The construction of an effective descriptor is achieved through the learning of multi-scale convolutional feature maps within a deep architecture. Ding et al. [227] developed a CNN-based model to extract high-level effective semantic features from RGB textured images obtained from using skeletal data. However, these methodologies have a significant amount of preprocessing steps and a chance to miss some effective information. Caetano et al. suggested SkeleMotion [228], which offers a novel skeleton image representation as an alternative input for neural networks to address these issues. Researchers have explored solutions to the challenge of long-time dependence, especially considering that CNNs do not extract long-distance motion information. To overcome this issue, Liu et al. [229] suggested a Subsequence Attention Network (SSAN) to improve the capture of long-term features. This network, combined with 3DCNN, uses skeleton data to record long-term features more effectively. Sun et al. [216] proposed a network for encoding 3D skeletal joint data into grayscale images and classifying human activities using a three-channel ResNet34-based fusion network, while noting potential limitations in handling occlusions and multi-person scenarios due to reliance on unobstructed single-person video inputs.

#### 4.4.2. RNN-LSTM-Based Methods

Approaches relying on Recurrent Neural Networks (RNNs) with LSTM [230,231] have garnered considerable popularity as a predominant DL methodology for skeleton-based action recognition. Moreover, these approaches have demonstrated exceptional proficiency in accomplishing video-based action recognition tasks [91,149,195,196,232,233]. The spatio-temporal patterns of skeletons exhibit temporal evolutions. Consequently, these patterns can be effectively represented by memory cells within the structure of RNN-LSTM models, as proposed by [230]. In a similar way, Du et al. [232] introduced a hierarchical RNN approach to capture the long-term contextual information of skeletal data. This involved dividing the human skeleton into five distinct parts based on its physical structure. Subsequently, each lower-level part was represented using an RNN, and these representations were then integrated to form the final representation of higher-level parts, which facilitated action classification. The problem related to gradient explosion and vanishing gradients occurs if the sequences are too long for actual training. To overcome this issue, Li et al. [234] suggested an Independent Recurrent Neural Network (IndRNN) to regulate gradient backpropagation over time, allowing the network to capture long-term dependencies. Shahroudy et al. [91] introduced a model for human action learning using a part-aware LSTM. This model involves splitting the long-term memory of the entire motion into part-based cells and independently learning the long-term context of each body part. The network’s output is then formed by combining the independent body part context information. Liu et al. [149] presented a spatio-temporal LSTM network named ST-LSTM, aimed at 3D action recognition from skeletal data. They proposed a technique called skeleton-based tree traversal to feed the structure of the skeletal data into a sequential LSTM network and improved the performance of ST-LSTM by incorporating additional trust gates. In their recent work, Liu et al. [233] directed their attention towards the selection of the most informative joints in the skeleton by employing a novel type of LSTM network called Global Context-Aware Attention (GCA-LSTM) to recognize actions based on 3D skeleton data. Two layers of LSTM were utilized in his study. The initial layer encoded the input sequences and produced a global context memory for these sequences. Simultaneously, the second layer carried out attention mechanisms over the input sequences with the support of the acquired global context memory. The resulting attention representation was subsequently employed to refine the global context. Numerous iterations of attention mechanisms were conducted, and the final global contextual information was employed in the task of action classification. Compared to the methodologies based on hand-crafted designed local features, the RNN-LSTM methodologies and their variations have demonstrated superior performance in the recognition of actions. Nevertheless, these methodologies tend to excessively emphasize the temporal information while neglecting the spatial information of skeletons [91,149,195,215,232,233]. RNN-LSTM methodologies continue to face difficulties in dealing with the intricate spatio-temporal variations of skeletal movements due to multiple issues, such as jitters and variability in movement speed. Another drawback of the RNN-LSTM networks [230,231] is their sole focus on modeling the overall temporal dynamics of actions, disregarding the detailed temporal dynamics. To address these limitations, in this investigation a CNN-based methodology can extract discriminative characteristics of actions and model the various temporal dynamics of skeleton sequences via the suggested Enhanced-SPMF representation, encompassing short-term, medium-term, and long-term actions.

#### 4.4.3. GNN or GCN-Based Methods

Graph Convolutional Neural Networks (GCNNs) are powerful DL-based methods designed to perform with non-Euclidean data. Unlike traditional CNNs and RNNs, which perform well with Euclidean data (such as images, text, and speech), they are unable to perform with non-Euclidean data [235,236,237,238,239,240]. The GCN was first introduced by Gori et al. [241] in 2005 to handle graph data. GCNNs with skeleton data enable spatial dependencies to be captured for accurate action recognition. The human skeleton data, consisting of joint points and skeletal lines, can be viewed as non-Euclidean graph data. Therefore, GCNs are particularly suited for learning from such data. There are two main branches of GCNs: Spectral GCN and Spatial GCN.

#### 4.4.4. Spectral GCN-Based Methods

Using and leveraging both eigenvalues and eigenvectors of the Graph Laplacian Matrix (GLM) to convert graph data from the temporal to the spatial domain [242], this model is not computationally efficient. To address this issue, Kipf et al. [243] enhanced the spectral GCN approach by allowing the filter operation of only one neighbor node to reduce the computational cost. While spectral GCNs have shown effectiveness in HAR tasks, their computational cost poses challenges when dealing with graphs.

#### 4.4.5. Spatial GCN-Based Methods

**Figure 10:**
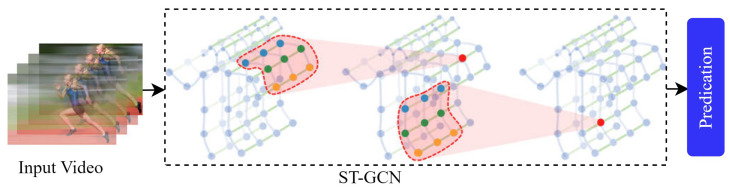
Skeleton-based HAR using ST-GCN [244].

Spatial GCN-based methods are more efficient in terms of computational than spectral GCNs. Therefore, spatial GCNs have become the main focus in many GCN-based HAR approaches due to efficiency. Yan et al. [244] developed the concept of ST-GCN, a model specifically designed for spatio-temporal data. As depicted in Figure 10, the ST-GCN, bodily joints (such as joints in a human skeleton) serve as the vertices in the graph while the edges denote the connection between the bodily bones within the same frame. Shi et al. [201] developed two-stream adaptive GCN models to improve the flexibility of graph networks. This model allows for the use of the end-to-end approach to learning the graph’s topology within the model. By adopting a data-driven methodology, the 2S-AGCN model becomes more adaptable to diverse data samples, increasing flexibility. Additionally, an attention mechanism is included to improve the robustness of the 2sAGCN model. For a further improvement to explore the enhancement of HAR methods, Shiraki et al. [245] proposed the spatio-temporal attentional graph (STA)-GCN to determine the challenge of varying the importance of joints across different human actions. Unlike traditional GCNs, STA-GCN takes into account both the significance and interrelationship of joints within the graph. Researchers have drawn inspiration from STA-GCN to further enhance GCN models [246,247]. For instance, the shift-GCN [248] model introduces the innovative shift-graph method to enhance the flexibility of the Spatio-Temporal Graph’s (STG) receptive domain. Additionally, the lightweight dot convolution technique is utilized to reduce the number of feature channels and make the model more efficient. Song et al. [249] present the residual-based GCN model to improve the performance of the model in terms of accuracy and computational efficiency for HAR. Similarly, Thakkar et al. [250] and Li et al. [251] presented methods to divide the human skeleton into separate body parts, and they developed the Partial-Based Graph Convolutional Network (PB-GCN) [250], which learns four subgraphs of the skeleton data. Li et al. [251] developed the Spatio-Temporal Graph Routing (STGR) scheme to better determine the connections between joints. Mehmood et al. [217] proposed EMS-TAGCN, a multi-stream adaptive GCN with spatial–temporal-channel attention for skeleton-based HAR, achieving good results across multiple datasets while recognizing increased model complexity and limited scalability without additional optimization. These methods help improve the segmentation of body parts for HAR. Table 5 summarizes the key comparative analysis of GCN variants in the skeleton-based HAR method, including findings and limitations.

### 4.5. Mathematical Derivation of the Skeleton-Based Learning Methods

For skeleton-based HAR, the given dataset is defined as:

(31) $$D=\left\{\left(S_{1},\ldots ,S_{n}\right),\left(y_{1},\ldots ,y_{n}\right)\right\}$$

where $S_{i}$ is the *i*-th training example representing a skeleton sequence and $y_{i}$ is the true label of the corresponding $S_{i}$. Each skeleton training example $S_{i}$ is represented as a spatio-temporal graph:

(32) $$S_{i}=\left(V_{i},E_{i}\right)$$

where $V_{i}$ is the set of nodes, $V_{i}=\left\{v_{1},v_{2},\ldots ,v_{n}\right\}$ is the set of nodes (keypoints), and *E* is the set of edges (spatial and temporal connections). Each node $v_{ti}$ represents a keypoint in the *t*-th frame, and each node has a feature vector $F\left(v_{ti}\right)\in R^{c}$ (e.g., 3D coordinates with or without confidence scores) [175]. The notation $F\left(v_{ti}\right)\in R^{c}$ refers to the feature vector for a node $v_{ti}$, which is a keypoint in the spatio-temporal graph for HAR. Each node represents a keypoint (joint) in the skeleton sequence, and $F(v_{ti})$ contains the characteristics describing that keypoint. Typically, these features include the 3D coordinates of the keypoint, (x,y,z), making $F\left(v_{ti}\right)\in R^{3}$. If confidence scores are included, $F\left(v_{ti}\right)\in R^{4}$, and additional features increase the dimensionality *c* accordingly.

#### 4.5.1. GCN

Let *G* be a graph representing the skeleton of a human body in each frame, and $G_{i}=S_{i}$ where $S_{i}=\left(V_{i},E_{i}\right)$. In addition, given the adjacency matrix *A*, which defines the spatial (intra-body) and temporal (inter-frame) relationships between nodes, the GCN layer update rule is:

(33) $$GCN_{out}=H^{\left(l+1\right)}=\sigma \left(\hat{A}H^{\left(l\right)}W^{\left(l\right)}\right)$$

where $H^{\left(l\right)}\in R^{N\times F_{l}}$ is the feature matrix at layer *l* with *N* nodes and $F_{l}$ features, $\hat{A}=D^{-(1/2)}\left(A+I\right)D^{-(1/2)}$ is the normalized adjacency matrix, $W^{\left(l\right)}\in R^{F_{l}\times F_{l+1}}$ is the learnable weight matrix at layer *l*, σ is the activation function (e.g., ReLU), and $H^{\left(l+1\right)}\in R^{N\times F_{l+1}}$ is the updated feature matrix. We can also write it as a GCN feature $GCN_{out}$.

#### 4.5.2. ST-GCN

To improve the efficiency of the feature, Yan et al. applied the ST-GCN model as a spatio-temporal graph, applying graph convolutions to learn both spatial and temporal relationships. The convolution is defined in [244] as ST-GCN; the convolution is applied over a spatio-temporal graph, where both the adjacency matrix (spatial relationships) and the temporal connections (connections between the same keypoints across time) are considered. So, the update rule for the ST-GCN layer becomes [244]:

(34) $$f_{\mathrm{out}}\left(v_{t,i}\right)=\sum_{v_{t,j}\in N\left(v_{t,i}\right)}\frac{1}{Z_{t,i}\left(v_{t,j}\right)}f_{\mathrm{in}}\left(v_{t,j}\right)\cdot w\left(l_{t,i}\left(v_{t,j}\right)\right)$$

We can define the equation as below to understand the spatial–temporal GCN rules.

(35) $${\mathrm{GCN}}^{\left(l+1\right)}=\sigma \left(D^{-(1/2)}\left(A+I\right)D^{-(1/2)}\;{\mathrm{GCN}}^{\left(l\right)}W^{\left(l\right)}\right)$$

Here $D^{-(1/2)}\left(A+I\right)D^{-(1/2)}$ is the normalized adjacency matrix, $W^{\left(l\right)}\in R^{F_{l}\times F_{l+1}}$ is the learnable weight matrix at layer *l*, σ is the activation function (e.g., ReLU). In addition A+I in the GCN equation corresponds to adding self-loops to the adjacency matrix to ensure that each node can aggregate information from itself. The degree matrix *D* normalizes the adjacency matrix to prevent nodes with high degrees from dominating the aggregation. To link the spatial–temporal convolution from ST-GCN [244] with the GCN update rule, Equation (33), we adapt the updated equation to consider both spatial and temporal dependencies, similar to the GCN framework. The spatial convolution within each frame is modeled by the adjacency matrix *A* and its normalization, whereas the temporal information is captured by connecting keypoints across frames. This can be done by modifying the adjacency matrix to include temporal connections (i.e., edges that link the same keypoints from consecutive frames). The final GCN-like update rule for ST-GCN, combining spatial and temporal dependencies, is:

(36) $${\mathrm{GCN}}^{\left(l+1\right)}=\sigma \left(D^{-(1/2)}\left(\left(A_{\mathrm{spatial}}+I\right)+A_{\mathrm{temporal}}\right)D^{-(1/2)}\;{\mathrm{GCN}}^{\left(l\right)}W^{\left(l\right)}\right)$$

where $A_{\mathrm{spatial}}$ captures the spatial relationships between keypoints within each frame, $A_{\mathrm{temporal}}$ captures the temporal relationships, linking the same keypoints across frames (i.e., between frame *t* and frame t+1), *I* is the identity matrix, ensuring that each node aggregates information from itself (self-loops), and m σ is the activation function (if needed, but you can omit it for a linear GCN).

#### 4.5.3. STA-GCN

STA-GCN enhances feature learning by adding spatial and temporal attention mechanisms. Spatial attention is applied as [245]:

(37) $${\hat{f}}_{s}=f_{s}\cdot \sigma \left(W_{s}\right)\;\mathrm{and}\;{\hat{f}}_{t}=f_{t}\cdot \sigma \left(W_{t}\right)$$

where $f_{s}$ is the feature matrix of the graph nodes, σ(·) is the sigmoid activation function, $W_{s}$ is the spatial attention weight matrix, $f_{t}$ is the feature vector at time step *t*, and $W_{t}$ is the temporal attention weight matrix. STA-GCN improves the feature learning process of the HAR by introducing attention mechanisms, which allow the model to focus on more relevant spatial and temporal features. This is particularly useful when certain key points or time steps have more significance than others in the HAR task, leading to better performance and robustness in dynamic environments.

#### 4.5.4. Shift-GCN

Shi et al. [201] introduces a simple shift operation to capture temporal dynamics efficiently:

(38) $$f_{\mathrm{shifted}}\left(v_{t,i}\right)=f_{\mathrm{in}}\left(v_{(t-\delta ),i}\right)$$

where $f_{shifted}\left(v_{ti}\right)$ is the shifted feature of node $v_{ti}$, $f_{in}\left(v_{(t-\delta )i}\right)$ is the input feature of node $v_{i}$ at time t−δ, and δ is the temporal shift offset. Shift-GCN offers a simple and computationally efficient way to capture temporal dynamics by shifting the node features in time. Unlike more complex models such as RNNs or LSTMs, Shift-GCN directly leverages prior time step information.

#### 4.5.5. InfoGCN

InfoGCN applies an information bottleneck to encourage compact, discriminative features. The loss is defined as [252]:

(39) $$\min_{q}E_{q(f|S)}\left[L(C\left(f;\theta_{c}\right),y)\right]+\beta I\left(S,f\right)$$

where q(f|S) is the variational distribution of features given skeleton graph *S*, L(·) is the loss function (e.g., cross-entropy), $C(f;\theta_{c})$ is the classifier with parameters $\theta_{c}$, *y* is the ground truth label, β is the trade-off weight for the mutual information term, and I(S,f) is the mutual information between input graph *S* and features *f*. InfoGCN uses an information bottleneck to encourage the model to learn compact and discriminative features. It balances the loss function with a mutual information term to optimize for compactness and relevance of features while retaining the ability to make accurate predictions.

#### 4.5.6. EMS-TAGCN

EMS-TAGCN adds an edge-motion stream that explicitly models joint-to-joint dynamics [253]:

(40) $$e_{ij}=v_{i}-v_{j}$$

where $e_{ij}$ is the relative motion feature between nodes *i* and *j* and $v_{i},v_{j}$ is the feature vectors of nodes *i* and *j*. EMS-TAGCN introduces an edge-motion stream that explicitly models joint-to-joint dynamics, improving the model’s ability to capture the motion between pairs of joints over time. This helps in understanding the relative motion between body parts in HAR. This survey explores the evolution of skeleton-based HAR models, examining various Graph Convolutional Networks (GCNs) such as GCN, ST-GCN, STA-GCN, Shift-GCN, InfoGCN, and EMS-TAGCN. Each model applies mathematical principles to learn spatial and temporal dependencies, enhance feature learning, and capture motion dynamics. For example, GCN applies capturing graph-based joint relationships between keypoints in each frame, as seen in Equation (33), while ST-GCN extends this to a spatio-temporal graph, incorporating both spatial adjacency and temporal connections (Equation (34)). The STA-GCN model introduces attention mechanisms (Equation (37)) to focus on relevant spatial and temporal features, improving performance in dynamic environments. Meanwhile, Shift-GCN simplifies temporal dynamics by shifting features over time (Equation (38)), improving computational efficiency. InfoGCN applies an information bottleneck (Equation (39)) to learn compact, discriminative features, while EMS-TAGCN models joint-to-joint dynamics by adding an edge-motion stream (Equation (40)) to capture relative motion between keypoints. Together, these models demonstrate how the field has evolved to tackle key challenges in skeleton-based HAR, such as learning adaptable structures, focusing on relevant features, and modeling subtle joint dynamics while acknowledging trade-offs like computational cost and sensitivity to noise.

## 5. Sensor-Based HAR

We summarize several publicly available datasets in Table 6, including year, sensor modalities, number of sensors, number of participants, number of activities, activity categories, and latest performance accuracy. Sensor-based HAR has gained significant attention due to the advancements in wearable technology and its applications in various domains. These include health monitoring, industrial safety, sports training, and more [254]. Unlike computer vision-based or Wi-Fi-based HAR, wearable sensors offer advantages such as privacy, user acceptance, and independence from environmental factors [255]. Challenges in sensor-based HAR include diverse data collection, handling missing values, and complex activity recognition. Wearable devices use sensors like accelerometers and gyroscopes to identify human activities, but both feature extraction and model training remain challenging. The challenges with machine learning approaches rely on manual feature extraction [256], while the DL approaches now enable automatic feature extraction from raw sensor data, leading to superior results [255]. Overall, sensor-based HAR holds promise for improving healthcare and safety [257,258].

Table 7 summarizes various existing works based on sensor modality for HAR using traditional ML and DL techniques, including the author name, year, datasets, modality sensor names, methods, classifier, and performance accuracy [259]. As mentioned in the table, researchers have enhanced HAR classification performance by improving ML feature engineering, and some researchers have developed advanced DL models like CNN and LSTM for automatic feature extraction. Most studies utilized datasets from multiple sensor types placed at different body positions.

**Table 6:** Databases for sensor modality.

Dataset Names	Year	Sensor Modalities	No. of Sensors	No. of People	No. of Activities	Activity Categories	Latest Performances
HHAR [260]	2015	Accelerometer, Gyroscope	36	9	6	Daily living activity, Sports fitness activity	99.99% [261]
MHEALTH [262]	2014	Accelerometer, Gyroscope, Magnetometer, Electrocardiogram	3	10	12	Atomic activity, Daily living activity, Sports fitness activity	97.83% [263]
OPPT [264]	2013	Acceleration, Rate of Turn Magnetic field, Reed switches	40	4	17	Daily living activity, Composite activity	100% [265]
WISDM [266]	2011	Accelerometer, Gyroscopes	1	33	6	Daily living activity, Sports fitness activity	97.8% [267]
UCIHAR [268]	2013	Accelerometer, Gyroscope	1	30	6	Daily living activity	
PAMAP2 [269]	2012	Accelerometer, Gyroscope, Magnetometer, Temperature	4	9	18	Daily living activity, Sports fitness activity, Composite activity	94.72% [270] 82.12% [265] 90.27% [267]
DSADS [271]	2010	Accelerometer, Gyroscope Magnetometer	45	8	19	Daily living activity, Sports fitness activity	99.48% [272]
RealWorld [273]	2016	Acceleration	7	15	8	Daily living activity, Sports fitness activity	95% [274]
Exer. Activity [275]	2013	Accelerometer, Gyroscope	3	20	10	Sports fitness activity	-
UTD-MHAD [88]	2015	Accelerometer, Gyroscope RGB camera, depth camera	3	8	27	Daily living activity, Sports fitness activity Composite activity Atomic activity	76.35% [276]
Shoaib [277]	2014	Accelerometer, Gyroscope	5	10	7	Daily living activity, Sports fitness activity	99.86% [278]
TUD [279]	2008	Accelerometer	2	1	34	Daily living activity, Sports fitness Composite activity	-
SHAR [280]	2017	Accelerometer	2	30	17	Daily living activity, Sports fitness activity Atomic activity	82.79% [281]
USC-HAD [282]	2012	Accelerometer, Gyroscope	1	14	12	Daily living activity, Sports fitness activity activity	97.25% [281]
Mobi-Act [283]	2016	Accelerometer, Gyroscope orientation sensors	1	50	13	Daily living activity, Atomic activity activity	75.87% [284]
Motion Sense [285]	2018	Accelerometer, Gyroscope	1	24	6	Daily living activity	95.35% [286]
van Kasteren [287]	2011	switches, contacts passive infrared (PIR)	14	1	10	Daily living activity Composite activity activity	-
CASAS [288]	2012	Temperature Infrared motion/light sensor	52	1	7	Daily living activity Composite activity activity	88.4% [289]
Skoda [290]	2008	Accelerometer	19	1	10	Daily living activity Composite activity activity	97% [291]
Widar3.0 [253]	2019	Wi-Fi	7	1	6	Atomic activity	82.18% [292]
UCI [268]	2013	Accelerometer, Gyroscope	2	30	6	Human activity	95.90% [270]
HAPT [293]	2016	Accelerometer, Gyroscope	1	30	12	Human activity	92.14% [270] 98.73% [278]

**Table 7:** Sensor data modality-based HAR models and performance.

Author	Year	Dataset Name	Modality Sensor Name	Methods	Classifier	Accuracy %
Jain et al. [294]	2017	UCI HAR	IMU Sensor	Fusion based	SVM, KNN	97.12
Ignatov et al. [295]	2018	WISDM UCI HAR	IMU Sensor	CNN	Softmax	93.32 97.63
Chen et al. [296]	2019	MHEALTH PAMAP2 UCI HAR	IMU	CNN	Softmax	94.05, 83.42 81.32
kavuncuouglu et al. [297]	2021	Fall and ADLs	Accelerometer Gyroscope Magnetometer	ML	SVM K-NN	99.96 95.27
Lu et al. [298]	2022	WISDM, PAMAP2 UCI-HAR	IMUs Accelerometers	CNN-GRU	Softmax	96.41 96.25 96.67
Kim et al. [299]	2022	WISDM USC-HAR	IMUs	CNN-BiGRU	Softmax	99.49 88.31
Lin et al. [300]	2020	Smartwach	Accelerometer gyroscope	Dilated CNN	Softmax	95.49
Nadeem et al. [301]	2021	WISDM PAMAP2 USC-HAD	IMU	HMM	Softmax	91.28 91.73 90.19
Zhang et al. [302]	2020	WiFi CSI	WiFi signal	Dense-LSTM	Softmax	90.0
Alawneh et al. [303]	2020	UniMib Shar WISDM	Accelerometer IMU Sensor	Bi-LSTM	Softmax	99.25 98.11
Wei et al. [304]	2024	WISDM PAMAP2 USC-HAD	IMU	TCN-Attention	Softmax	99.03 98.35 96.32
Yao et al. [281]	2024	PAMAP2 USC-HAD, UniMiB-SHAR OPPORTUNITY	IMUs Accelerometers	ELK ResNet	Softmax	95.53 97.25 82.79 87.96
El-Adawi et al. [263]	2024	MHEALTH	IMU	GAF+ DenseNet169	Softmax	97.83
Sarkar et al. [305]	2023	UCI-HAR WISDM, MHEALTH PAMAP2 HHAR	IMUs Accelerometers	CNN with GA	SVM	98.74 98.34 99.72 97.55 96.87
Semwal et al. [306]	2023	WISDM PAMAP2 USC-HAD	IMUs	CNN and LSTM	Softmax	95.76 94.64 89.83
Zhang et al. [278]	2024	DSADS HAPT	IMU	Multi-STMT	Softmax	99.86 98.73
Saha et al. [286]	2024	UCI HAR Motion-Sense	IMU	FusionActNet	Softmax	97.35 95.35
Liu et al. [307]	2025	UCI-HAR WISDM	Accelerometer Gyroscope	UC Fusion	Softmax	96.84 98.85
Khan et al. [308]	2025	HAPT Human activities	AAccelerometer Gyroscope	1D-CNN + LSTM	Softmax	97.84 99.04
sarakon et al. [309]	2025	WISDM DaLiAc MotionSense PAMAP2	Accelerometer	MLP	Softmax	95.83 97.00 94.65 98.54
Yao et al. [310]	2025	PAMAP2 WISDM USC-HAD	Accelerometer Gyroscope Magnetometer	MLKD	Softmax	92.66 98.22 95.42
Thakur et al. [311]	2025	UCI-HAR WISDM OPPORTUNITY HAR	Accelerometer Gyroscope Magnetometer GPS	CNN + RNN	Softmax	96 95 93 95
Hu et al. [312]	2025	UCI-HAR HAPT RHAR	Accelerometer Gyroscope Magnetometer GPS	AResGAT-LDA	Softmax	96.62 94.56 85.08
Yu et al. [313]	2025	UCI-HAR USC-HAD WISDM DSADS	Accelerometer Gyroscope Magnetometer GPS	ASK-HAR	Softmax	97.25 89.40 98.46 89.42
Muralidharan et al. [314]	2025	MobiAct	Accelerometer Gyroscope Orientation sensors	CNN-RNN	Softmax	94.69
Yang et al. [315]	2025	UCI-HAR RealWorld MotionSense	Accelerometer Gyroscope	Semi-supervised	Softmax	97.5 95.6 94.2
Ye et al. [265]	2024	OPPT, PAMAP2	IMU	CVAE-USM	GMM	100 82.12
Kaya et al. [267]	2024	UCI-HAPT WISDM, PAMAP2	IMU	Deep CNN	Softmax	98 97.8 90.27
Zhang et al. [272]	2024	Shoaib, SisFall HCIHAR, KU-HAR	IMU	1DCNN-Att -BiLSTM	SVM	99.48 91.85 96.67 97.99
Sharen et al. [316]	2024	WISDM UCI-HAR KU-HAR	Accelerometer Gyroscope	WISNet	Softmax	96.41 95.66 94.01
Teng et al. [317]	2025	UCI-HAR PAMAP2 UNIMIB-SHAR USC-HAD	Accelerometer Gyroscope Magnetometer	CNN-TSFDU-LW	Softmax	97.90 94.34 78.90 94.71
Dahal et al. [318]	2025	mHealth UCI-HAR WISDM	Accelerometer Gyroscope Magnetometer	Stack-HAR	Gradient Boosting	99.49 96.87 90.00
Pitombeira-Neto et al. [319]	2025	PAMAP2 USC-HAD	Accelerometer Gyroscope Magnetometer	BDLM	Bayesian updating	96.00

### 5.1. Preprocessing of the Sensor Dataset

Preprocessing sensor data is crucial for reliable analysis and effective maintenance. Consequently, data collected from sensing devices must be preprocessed before being utilized for any analysis. Poor data quality, including missing values, outliers, and spikes, can impact the performance results. Preprocessing steps like imputing missing data, noise reduction, and normalization are significant. A fast, scalable module is needed for real-time data preprocessing, especially in predictive maintenance systems [320]. After preprocessing the sensor data, the second step is feature engineering, which involves creating new characteristics from existing data. Its main goals are to improve connections between input and output variables in forecasting models and to select the most useful features, enhancing model quality and efficiency. Finally, a proper model must be designed and implemented.

### 5.2. Sensor Data Modality Based HAR System Using Feature Extraction with Machine Learning

Previous studies on sensor-based HAR have involved manually extracting features from raw sensor data and using conventional ML techniques like SVM, Random Forest, KNN, Decision Tree, and NB [321,322,323]. Kavuncuoglu et al. [297], by combining accelerometer and magnetometer data with SVM, improved fall and activity classification. Feature-level fusion has outperformed fraction-level fusion with multiclass SVM and KNN classifiers on UCI HAR and physical activity sensor datasets. Using EEG data, models like RF and GB demonstrated excellent performance [294], with Local Interpretable Model-agnostic Explanations (LIMEs) providing insights into significant EEG features [324]. Introducing new activity classifications and novel feature engineering with models like Random Forest, KNN, and SVM has enhanced activity recognition accuracy. However, these traditional methods depend heavily on the quality of feature engineering, requiring domain-specific expertise to extract and select relevant features, which may not generalize across all activities [325]. Yang et al. [315] developed a semi-supervised learning framework for HAR that employs Difference Alignment Contrastive Loss (DAC Loss) to align individual differences in data, improving model performance while recognizing challenges in generalization across various sensor configurations and environments. Dahal et al. [318] proposed the Stack-HAR framework, an ensemble learning approach that improves HAR by stacking multiple models and using a metalearner. The framework’s performance on the WISDM dataset was slightly lower due to the reliance on accelerometer data alone, which limits the granularity needed for dynamic activities like walking. Pitombeira-Neto et al. [319] presented an ensemble Bayesian Dynamic Linear Model (BDLM) for HAR, which is efficient, requires minimal data preprocessing, and operates online. The approach is demonstrated to perform competitively with other benchmark methods, using two real-world datasets, PAMAP2 and USC-HAD. Their method’s performance can be impacted by a large number of users in the ensemble, leading to high computational times.

### 5.3. Sensor Data Modality-Based HAR System Using a Deep Learning Approach

Recently, many researchers have developed DL-based methods for HAR using sensor-based datasets, such as CNNs and RNNs, which automatically learn complex features from raw sensor data without manual feature extraction. These models achieve state-of-the-art results in HAR. However, CNNs may not capture time-domain characteristics effectively. Recently, Hu et al. [312] introduced AResGAT-LDA, a residual graph attention network integrating linear discriminant analysis and adversarial learning for semi-supervised HAR, addressing label scarcity and robustness, though it requires significant computational power for training on high-dimensional data.

#### 5.3.1. Background of the Deep Learning-Based Temporal Modeling TCN

Recently, many studies have revolved around advancements in HAR using ambient sensors. It highlights the integration of various types of sensors—user-driven, environment-driven, and object-driven—into HAR systems [289]. Recent progress in HAR involves leveraging DL-based techniques, including Transformer models with multi-head attention mechanisms, to effectively capture temporal dependencies in activity data [24]. Additionally, the importance of sensor frequency information and the analysis of time and frequency domains in understanding sensor-driven time series data are emphasized [326]. The previous approach aims to address challenges such as adapting HAR systems to new activities in dynamic environments [255]. Kim et al. [289] developed a contrastive learning-based novelty detection (CLAN) method for HAR from sensor data. They addressed challenges like temporal and frequency features, complex activity dynamics, and sensor modality variations by leveraging diverse negative pairs through data augmentation. The two-tower model extracts invariant representations of known activities, enhancing recognition of new activities, even with shared features. Wei et al. [304] presented a Time Convolution Network with Attention Mechanism (TCN-Attention-HAR) model designed to enhance HAR using wearable sensor data. Addressing challenges such as effective temporal feature extraction and gradient issues in deep networks, the model optimizes feature extraction with appropriate temporal convolution sizes and prioritizes important information using attention mechanisms. Zhang et al. [272] presented Multi-STMT, a multilevel model for HAR using wearable sensors that integrate spatio-temporal attention and multiscale temporal embedding; the model combines CNN and BiGRU modules with attention mechanisms to capture nuanced differences in activities. Yu et al. [313] introduced ASK-HAR, a DL method using attention-based multi-core selective kernel convolution and CBAM to capture multiscale sensor data features for accurate HAR, though it faces challenges with stationary activity recognition and deployment in complex environments. In a recent study, ref. [327] presented a DL-based domain adaptation framework specifically designed for time-series sensor data in cross-user HAR. Teng et al. [317] proposed the CNN-TSFDU-LW, a novel model designed for sensor-based HAR, integrating the dual-decoupling of temporal and spatial features along with layer-wise training, which enhances computational efficiency due to the increased Memory Access Cost (MAC) with the layer-wise approach.

#### 5.3.2. CNN-Based Various Stream for HAR

Ignatov et al. [295] utilized a DL-based approach for real-time HAR with mobile sensor data. They employed a CNN for local feature extraction and integrated simple statistical features to capture global time series patterns. The experimental evaluations of the WISDM and UCI datasets demonstrate high accuracy across various users and datasets, highlighting their effectiveness in the DL-based method without needing complex computational resources or manual feature engineering. Chen et al. [296] developed a semi-supervised DL-based model for imbalanced HAR that utilized multimodal wearable sensory data. Addressing challenges such as limited labeled data and class imbalance, the model employs a pattern-balanced framework to extract diverse activity patterns. They used recurrent convolutional attention networks to identify salient features across modalities. Kaya et al. [267] presented a 1D-CNN-based approach for accurate HAR from sensor data. They evaluated their model using raw accelerometer and gyroscope sensor data from three public datasets: UCI-HAPT, WISDM, and PAMAP2. Zhang et al. [291] presented a method, HAR, using a sensor data modality called ConvTransformer. They combined CNN, Transformer, and attention mechanisms to handle the challenge of extracting both detailed and overall features from sensor data. Liu et al. [307] proposed UC Fusion, a DL method for HAR that combines unique and common features from multiple wearable sensors, achieving superior accuracy on the UCI HAR and WISDM datasets compared to existing methods. Sarakon et al. [309] proposed a multisource data fusion approach for HAR using a Multi-Layer Perceptron (MLP), achieving high accuracy across diverse datasets while recognizing limitations in generalization due to sensor placement variability and insufficient evaluation in resource-constrained or real-time environments. Yao et al. [310] proposed a novel multi-teacher knowledge distillation framework, MLKD, using long-kernel CNNs to transfer rich spatio-temporal knowledge to compact student networks for sensor-based HAR, while noting limitations in automatic kernel size selection and privacy concerns in real-world deployments. Park et al. [328] proposed HT-AggNet, a novel deep neural architecture with hierarchical temporal aggregation and near-zero-cost layer stacking for efficient HAR, achieving results across diverse datasets while noting potential challenges in optimal depth tuning and broader deployment on constrained devices. Sharen et al. [316] proposed a novel deep learning model, WISNet, leveraging a custom 1D-CNN architecture with specialized blocks like channel attention for enhanced HAR.

#### 5.3.3. RNN, LSTM, Bi-LSTM for HAR

In most of the recent work, including RNNs [329] plays a crucial role in handling temporal dependencies in sensor data for HAR. To address challenges like gradient issues, LSTM networks were developed [330]. Researchers [272,302,303,331] have also explored attention-based BiLSTM models, achieving the best performance compared to other DL-based methods. The experimental evaluations on various datasets shown in Table 7 demonstrate high accuracy across various users and datasets, highlighting their effectiveness in the DL-based method without needing complex computational resources or manual feature engineering. Saha et al. [286] presented Fusion ActNet, an advanced method for HAR using sensor data. It features dedicated residual networks to capture static and dynamic actions separately, alongside a guidance module for decision-making, through a two-stage training process and evaluations on benchmark datasets. The authors [314] proposed a hybrid CNN Bi-LSTM model for HAR using sensor data, achieving high classification accuracy while acknowledging challenges in differentiating similar fall-related activities. Murad et al. [330] used Deep Recurrent Neural Networks (DRNNs) in HAR, highlighting their ability to capture long-range dependencies in variable-length input sequences from body-worn sensors. Unlike traditional approaches that overlook temporal correlations, DRNNs, including unidirectional, bidirectional, and cascaded LSTM frameworks, perform well on diverse benchmark datasets. They perform the comparison of conventional machine learning approaches like SVM and KNN, as well as other deep learning techniques such as DBNs and CNNs, demonstrating their effectiveness in activity recognition tasks.

#### 5.3.4. Integration of CNN and LSTM-Based Technique

Several studies have developed hybrid models that utilize and combine different DL architectures and can report high-performance accuracy in HAR. For instance, a hybrid CNN-LSTM model [152,306,332] improved sleep–wake detection using heterogeneous sensors. Additionally, designs like TCCSNet [333] and CSNet leverage temporal and channel dependencies to enhance human behavior detection. Ordonez et al. [329] developed a model for HAR using CNN and LSTM recurrent units. They extract features from raw sensor data, support multimodal sensor fusion, and model complex temporal dynamics without manual feature design. Evaluation of benchmark datasets, such as Opportunity and Skoda, shows significant performance improvements over traditional methods, highlighting their effectiveness in HAR applications. Zhang et al. [334] developed a multi-channel DL-based network called a hybrid model (1DCNN-Att-BiLSTM) for improved recognition performance, evaluation using publicly accessible datasets, and comparison with ML and DL models. El-adawi et al. [263] developed a HAR model within a Wireless Body Area Network (WBAN). The model leverages the Gramian Angular Field (GAF) and DenseNet. By converting time series data into 2D images using GAF and integrating them with DenseNet, they achieved good performance accuracy. Khan et al. [308] proposed an ensemble 1D-CNN and LSTM model for transition-aware HAR using wearable sensor data, demonstrating high accuracy across both postural and dynamic activities, but recognizing limitations in model simplification for deployment on resource-constrained microdevices. Cemiloglu et al. [335] presented a compact hybrid LSTM-CNN model for HAR using wearable sensor data, effectively mitigating data heterogeneity across devices and environments, though it was constrained by challenges in layer configuration, sensor variability, and cloud-based deployment limitations. Thakur et al. [311] proposed a hybrid CNN–RNN model optimized with a GWO–WOA feature selection method for sensor-based HAR, achieving good results across benchmark datasets while facing limitations in scalability, high training complexity, and limited activity diversity.

### 5.4. Radio Frequency (RF)-Based HAR Techniques

**Figure 11:**
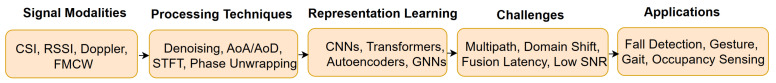
Taxonomy of RF-based HAR.

Figure 11 presents a structured knowledge map of RF-based HAR. It outlines the key components of the system pipeline and categorizes the associated research challenges and applications. In the following subsection, we provide a detailed description of RF-based HAR techniques, including RF-based data preprocessing, filtering, denoising, and feature extraction methods, as well as classification techniques.

#### 5.4.1. RF Dataset and Signal Acquisition

HAR with RF signals typically begins with data from devices like Wi-Fi Network Interface Cards (NICs) (e.g., Intel 5300), USRP software-defined radios, or radar systems. These provide raw measurements including Channel State Information (CSI), Received Signal Strength Indicator (RSSI), and sometimes phase or frequency shift data [336]. CSI, the most widely used, captures amplitude and phase variations across antennas and subcarriers, enabling detailed human motion analysis [336,337].

#### 5.4.2. Filtering, Denoising, and Segmenting the Signal

RF signals are inherently noisy due to multipath fading and hardware imperfections. Common denoising methods include Hampel filters for outlier removal [337,338], Butterworth filters for high-frequency noise [339], Kalman filters for motion estimation, and wavelet thresholding for time-frequency denoising, along with segmentation methods like amplitude variance, energy thresholds, Doppler spectrogram peaks, and LOF identify activity boundaries [337].

#### 5.4.3. Multipath Effects and Mitigation Techniques

Multipath propagation remains a persistent and fundamental challenge in RF-based HAR, particularly within indoor environments. When radio signals encounter obstacles such as walls, furniture, or human bodies, they reflect, scatter, and diffract, creating multiple propagation paths that interfere constructively and destructively at the receiver. This results in distortion of the CSI, particularly in the amplitude and phase domains, which in turn complicates the extraction of stable and discriminative features for activity classification [340,341]. Several mitigation strategies have been proposed in the recent literature to improve signal fidelity and model robustness:

- Angle-of-Arrival (AoA) and Angle-of-Departure (AoD) Estimation: Methods such as MUSIC and ESPRIT leverage antenna array processing to spatially resolve signal paths, helping isolate the Line-of-Sight (LoS) component from multipath reflections [340].
- Time-Frequency Analysis: Doppler spectrograms and Short-Time Fourier Transform (STFT) techniques decompose CSI into time-varying frequency components, making it easier to detect motion-induced frequency shifts associated with human activities [341].
- Graph-Based Path Modeling: By jointly estimating parameters like AoA, Time-of-Flight (ToF), and Doppler shifts, recent approaches construct graph-based signal representations to maintain spatial–temporal consistency in dynamic scenarios.
- Phase Unwrapping and CSI Denoising: Techniques including Hilbert transforms, wavelet filtering, and statistical smoothing are applied to reduce high-frequency noise and unwrap distorted phase responses, enhancing the usability of CSI features.
- Domain Adaptation: DL models trained in one environment often fail to generalize to others due to the environmental sensitivity of RF signals. Adversarial domain adaptation methods (e.g., DANN, GAN-based transfer learning) have shown promise in aligning latent representations across domains [340].

Despite these advances, accurately modeling and compensating for multipath effects in real-time and across varying conditions remains an open research problem. Real-world systems such as SiFall [341] demonstrate that combining denoising, unsupervised learning, and anomaly detection can yield effective results, but general-purpose, environment-agnostic solutions are still lacking.

#### 5.4.4. RF Feature Extraction

Feature extraction plays a crucial role in RF-based HAR by transforming raw signal data into meaningful representations. These features are typically grouped into four categories [337]. Time-domain features, such as mean, standard deviation, interquartile range, and energy, are simple to compute and commonly used in many systems. Frequency-domain features are used to analyze periodic patterns using the Fast Fourier Transform (FFT), from which dominant frequencies, power spectral density, and spectral entropy can be extracted—these are especially effective for detecting repetitive activities like walking or running. Time-frequency features provide a joint representation of temporal and spectral changes using methods like Discrete Wavelet Transform (DWT) and Hilbert–Huang Transform (HHT), allowing accurate recognition of dynamic or fast-changing gestures. Finally, spatial features such as Angle of Arrival (AoA) and Time of Flight (ToF) are used in systems equipped with antenna arrays or radar sensors; they enable motion tracking and localization by estimating direction and distance of movement. These categories of features offer different perspectives on human motion and are chosen based on the hardware setup and the nature of the activity being monitored.

#### 5.4.5. Classification

After preprocessing the RF signals, the extracted features are classified into human activity labels using different methods [337]. The main approaches include template matching, ML, and DL. ML techniques like SVM and KNN require training with labeled data and are effective for recognizing multiple activities across different environments. DL approaches, including CNNs and LSTM networks, automatically learn feature representations from data and can handle complex patterns with high accuracy. Still, they require larger datasets and longer training times. The choice of classification method depends on factors like activity complexity, training data availability, and system requirements.

#### 5.4.6. ML and DL Methods for RF-Based Datasets

Recent RF-based HAR approaches leverage diverse modeling techniques, including CSI temporal dynamics, Doppler spectrograms, and domain adaptation, to enhance motion and activity recognition [342,343,344,345]. For example, Uysal et al. [346] achieved over 99% accuracy with variance features and Decision Trees; Li et al. [343] integrated Doppler spectrograms with LSTM models (91.3%); Zhao et al. [342] used RFID with GCNs (92.8%); and Saeed et al. [347] achieved up to 97% with USRP-based tree classifiers. Generative models like RF-AIGC [348], as well as CNN-based approaches such as HAR-SAnet [349] and TARF [350], address domain adaptation and modality variation challenges. Multimodal fusion (e.g., Wi-Fi CSI with IMU [351] or Doppler with audio [352]) further improves robustness. Exploratory work on OTFS waveforms at 60GHz mmWave [345] shows potential but lacks quantitative metrics. Overall, ML models like SVMs and Random Forests require carefully engineered features to remain robust, while DL models (CNNs, LSTMs) excel at learning complex patterns but require large labeled datasets and face domain shift challenges [337].

### 5.5. Mathematical Derivation of the Sensor-Based Learning Method

In the case of sensor-based HAR, suppose the given dataset is defined as:

(41) $$D=\left\{\left(X_{1},\ldots ,X_{n}\right),\left(y_{1},\ldots ,y_{n}\right)\right\}$$

where $X_{i}$ is the *i*-th training example representing a time-series sequence of sensor readings and $y_{i}$ is the true label of the corresponding $X_{i}$. Each training example $X_{i}$ can be represented as a matrix with dimensions (T,s), where *T* is the number of time steps or sensor samples and *s* is the number of sensor channels or modalities (e.g., accelerometer axes, gyroscope, etc.). Training on a single modality for sensor data can be written as the following equation:

(42) $$L\left(C\left(\phi_{m}\left(X_{i};\theta_{m}\right);\theta_{c}\right),y_{i}\right)$$

where $\phi_{m}$ is a deep neural network feature extractor designed for time-series sensor data (such as a 1D-CNN, RNN, or Transformer) with parameters $\theta_{m}$. *C* is a classifier with parameters $\theta_{c}$. *L* is the loss function measuring the discrepancy between the predicted label and the true label $y_{i}$. This formulation allows sensor-based HAR models to effectively capture temporal dependencies in human motion signals by processing the time-series data. State-of-the-art models extend this framework by incorporating techniques such as temporal convolutions, recurrent architectures, and attention mechanisms to enhance learning from noisy and multimodal sensor inputs.

## 6. Multimodal Fusion Modality-Based Action Recognition

Action recognition through the utilization of a dataset that consists of multiple modalities necessitates the act of discerning and categorizing human actions or activities. This dataset encompasses various forms of information, including visual, audio, and sensor data. Integrating diverse sources of information within multimodal datasets affords a better comprehension of actions. From the perspective of the input data’s modality, DL techniques can acquire human action characteristics through a diverse range of modal data. Similarly, the ML-based algorithm aims to process the information from multiple modalities. By using the strengths of various data types, multimodal ML can often perform more accurate HAR tasks. There are several types of multi-modality learning methods, including fusion-based methods such as RGB with skeleton and depth-based modalities. Generally, fusion refers to combining the information of two or more modalities to train the model and provide accurate results of HAR. There are two main approaches widely utilized in multi-modality fusion schemes, namely, score fusion and feature fusion. The fusion-based approach combines scores obtained from various sources, including weight averaging [353] or learning a score fusion [354] model, while the feature fusion [355] focuses on integrating features extracted from different modalities. Ramani et al. [356] developed an algorithm that combines depth image and 3D joint position data using local spatio-temporal features and dominant skeleton movements and trains a Random Decision Forest (RDF). Researchers have increasingly explored DL techniques to extract action-effective features utilizing RGB, depth, and skeleton data. These methods facilitate multimodal feature learning from deep networks [22,23,47,149,357], encompassing appearance image information such as optical flow sequences, depth sequences, and skeleton sequences. DL networks are proficient at learning human action effective features by performing single-modal data or multimodal fusion data [185,358,359]. Note that score fusion and feature fusion are important in advancing HAR technology to provide accurate results. A recent study [360] presents a robust tri-modal DL architecture combining CNNs and attention mechanisms, significantly enhancing HAR for home-based rehabilitation using RGB and skeleton-based data and Continuous Wavelet Transform representations. One study [361] introduces a multi-input CNN framework that enhances HAR by fusing spectrograms, recurrence plots, and multi-channel plots from accelerometer data, achieving high accuracy without complex preprocessing. CIR-DFENet [362] fuses time-series and image-based representations from tri-axial accelerometer data using a dual-stream DL network with attention mechanisms, achieving 99.4% accuracy in classifying complex gymnastic activities. Additionally, we provided the most popular benchmark HAR datasets, which come from the multimodal fusion dataset, which is demonstrated in Table 8. The dataset table presents details of the datasets, including modalities, creation year, number of classes, number of subjects who participated in recording the dataset, number of samples, and the latest performance accuracy of the dataset with citation.

### 6.1. Multimodal Fusion-Based HAR Dataset

**Figure 12:**
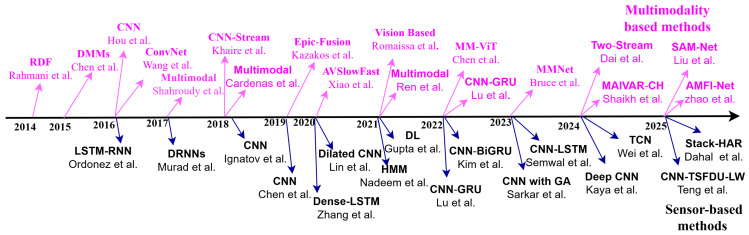
Milestone approaches for HAR. The pink font is the multi-modality-based methods [51,84,111,177,298,356,362,363,364,365,367,370,371,373,377,378,380,386] and the black font is the sensor-based methods [267,295,296,299,300,301,302,304,305,306,317,318,329,330,331].

Figure 12 demonstrates the year-wise end-to-end deep learning method developed by various researchers for sensor and multimodal fusion-based HAR systems. Liu et al. [373] proposed SAM-Net, a semantic-assisted multimodal network that integrates skeleton, RGB video, and text modalities for superior action recognition performance on RGB-D videos. Xefteris et al. [374] proposed a novel multimodal fusion method for 3D human pose estimation, combining visual data from a single RGB camera with sensor data from six IMUs, using a hybrid LSTM-Random Forest network, and reported good accuracy. Table 9 presents a comprehensive overview of benchmark datasets for HAR using various modalities. The datasets include combinations of RGB, skeleton, depth, infrared, acceleration, and gyroscope data, providing rich and diverse sources for model training and evaluation. For instance, the MSRDailyActivity3D dataset, introduced in 2012, includes RGB, skeleton, and depth data, featuring 16 classes, 10 subjects, and 320 samples with a notable accuracy of 97.50% [83,84].

These datasets are crucial for advancing HAR research, offering extensive and varied data for developing robust and accurate models.

**Table 9:** Multi-modality fusion-based HAR benchmark datasets.

Dataset	Data Set Modalities	Year	Class	Subject	Sample	Latest Accuracy
MSRDaily Activity3D [83]	RGB, Skeleton, Depth	2012	16	10	320	97.50% [84]
N-UCLA [85]	RGB, Skeleton, Depth	2014	10	10	1475	99.10% [86]
Multi-View TJU [87]	RGB, Skeleton, Depth	2014	20	22	7040	-
UTD-MHAD [88]	RGB, Skeleton, Depth, Acceleration, Gyroscope	2015	27	8	861	95.0% [89]
UWA3D Multiview II [90]	RGB, Skeleton, Depth	2015	30	10	1075	-
NTU RGB+D [91]	RGB, Skeleton, Depth, Infrared	2016	60	40	56,880	97.40% [86]
PKU-MMD [92]	RGB, Skeleton, Depth, Infrared	2017	51	66	10,076	94.40% [93]
NEU-UB [94]	RGB, Depth	2017	6	20	600	-
Kinetics-600 [95]	RGB, Skeleton, Depth, Infrared	2018	600	-	595,445	91.90% [71]
RGB-D Varing-View [96]	RGB, Skeleton, Depth	2018	40	118	25,600	-
Drive&Act [98]	RGB, Skeleton, Depth	2019	83	15	-	77.61% [99]
MMAct [100]	RGB, Skeleton, Acceleration, Gyroscope	2019	37	20	36,764	98.60% [101]
Toyota-SH [102]	RGB, Skeleton, Depth	2019	31	18	16,115	-
IKEA ASM [103]	RGB, Skeleton, Depth	2020	33	48	16,764	-
ETRI-Activity3D [104]	RGB, Skeleton, Depth	2020	55	100	112,620	95.09% [105]
UAV-Human [106]	RGB, Skeleton, Depth	2021	155	119	27,428	55.00% [107]

### 6.2. Fusion of RGB, Skeleton, and Depth Modalities

Recently, several hand-crafted feature-based approaches [94,375] have been developed to explore multi-modalities such as RGB, skeleton, and depth to improve the performance of the action recognition tasks, while the DL-based approaches [84,376,377,378] have been proposed due to them providing good performance. Shahoudy et al. [84] studied and explored the concept of correlation analysis between the different modalities and factorized them into desired independent components. They used a structured sparse classifier for the HAR task. Hu et al. [376] analyzed the time-varying information across the fusion of multi-modality, such as RGB, skeleton, and depth-based. They extracted temporal features from each modality and then concatenated them along the desired modality dimension. These multimodal temporal features were then input into the model. Khaire et al. [377] developed a CNN network with five streams. These streams take inputs from MHI [126], DMM [219], and skeleton images generated from RGB, depth, and skeleton sequences. Each CNN stream was trained separately, and the final classification scores were obtained by combining the output scores of all five CNN streams by utilizing a weighted product model. Similarly, Khair et al. [379] used a fusion of three methods to merge skeletal, RGB, and depth modalities. Cardenas et al. [378] utilized three distinct optical spectra channels from skeleton data [380] and dynamic images from RGB and depth videos. These features were fed into a pre-trained CNN to extract multimodal features. Finally, Hou et al. [380] used a feature aggregation module for classification tasks. Table 10 compares HAR performance across different modalities on unified datasets. Results consistently show that single-modality methods (e.g., skeleton, RGB, or depth) achieve lower accuracy (e.g., 69.90% to 80.30% on NTU RGB+D) compared to multimodal fusion methods, which reach significantly higher accuracies (e.g., up to 95.82% on NTU RGB+D). This highlights the benefit of integrating multiple modalities for improved recognition performance. For instance, Liu et al. [381] reported high performance using skeleton and RGB data on NTU-60, PKU-MMD, and N-UCLA datasets (98.0%, 98.0%, and 90.8%, respectively). Liu et al. [373] further improved performance by integrating text data (e.g., action labels or semantic information) alongside skeleton and RGB, achieving 98.5%, 98.4%, and 92.3%, respectively. This demonstrates that adding semantic information to visual and skeletal modalities can further enhance recognition in challenging scenarios.

### 6.3. Fusion of Signal and Visual Modalities

Signal data complements visual data by providing additional information. Various DL-based approaches have been proposed to merge these modalities for HAR. Wang et al. [385] proposed three-stream CNN models to extract features from multi-modalities. They evaluated the performance of both feature fusion and score fusion, with feature fusion showing superior performance. TSN [51] showed improved performance and Kazakos et al. [386] introduced the Temporal Binding Network (TBN) for egocentric HAR, integrating audio, RGB, and optical flow inputs. TBN utilized a three-stream CNN to merge these inputs within each Temporal Binding Window, enhancing classification through temporal aggregation. Their findings demonstrated TBN’s superiority over TSN [51] in audio–visual HAR tasks. Additionally, Gao et al. [387] utilized audio data to minimize temporal redundancies in videos, employing knowledge distillation from a teacher network trained on video clips to a student network trained on image–audio pairs for efficient HAR. Xiao et al. [177] developed a novel framework combining audio and visual information, incorporating slow and fast visual pathways alongside a faster audio pathway across multiple layers. They employed two training strategies: randomly dropping the audio pathway and hierarchical audio–visual synchronization, facilitating the training of audio-video integration. In addition, in multimodal HAR-based approaches such as Bruce et al. [367], a multimodal network (MMNet) fuses skeleton and RGB data using a spatio-temporal GNN to transfer attention weights, significantly improving HAR accuracy, while Venkatachalam et al. [388] proposed a hybrid 1D CNN with an LSTM classifier for HAR. Yu et al. [389] introduced DANet, a dual-attention-enabled DL model for cognitive workload recognition using multimodal EEG and eye-tracking data. Overall, the objective of data fusion methods is to capitalize on the benefits of integrating various datasets to achieve a more robust and comprehensive feature representation. Consequently, the central issue that arises in the development of most data-fusion-based techniques revolves around determining the most efficient manner in which to combine disparate data types. This is typically addressed by employing conventional early and late fusion strategies. The initial fusion occurs at the feature level, involving feature concatenation as the input to the recognition model. In contrast, the latter scenario performs fusion at the score level, integrating the output scores of the recognition model with diverse data types. The multimodal data fusion methods generally yield better recognition results than single-data approaches. However, the multimodal data fusion methods approach requires processing larger datasets and dealing with higher feature dimensions, thereby increasing the computational complexity of action recognition algorithms.

### 6.4. Mathematical Derivation of the Multimodal Learning Methods

In multimodal learning, multiple types of data (modalities) are combined to improve model performance. Let $X_{i}^{\left(m\right)}$ denote the *i*-th training sample from modality *m*, where m∈{1,2,…,M}. Each modality has its own deep neural network feature extractor:

(43) $$\phi_{m}\left(X_{i}^{\left(m\right)}\right)$$

where $\phi_{m}\left(\cdot \right)$ represents the CNN-based (or Transformer-based) encoder for modality *m*. For single-modality training (e.g., RGB), the learning objective can be defined as:

(44) $$L\left(C\left(\phi_{m}\left(X_{i}^{\left(m\right)}\right)\right),y_{i}\right)$$

where L(·) denotes the loss function (e.g., cross-entropy), C(·) is the classifier network that maps extracted features to predicted labels, and $y_{i}$ is the ground truth label for sample *i*. When dealing with multiple modalities, the features from these modalities can be fused using an operator ⊕ (such as concatenation or weighted sum) [175]:

(45) $$L_{multi}=L\left(C\left(\phi_{audio}⊕\phi_{video}\right),y_{i}\right)$$

where $\phi_{audio}$ and $\phi_{video}$ are the extracted features from the audio and video modalities, respectively. For generalized multimodal learning, the extracted features from all *M* modalities can be fused:

(46) $$\phi_{fuse}\left(X_{i}\right)=\overset{M}{\underset{m=1}{⨁}}\phi_{m}\left(X_{i}^{\left(m\right)}\right)$$

where $\phi_{fuse}\left(\cdot \right)$ represents the fusion operation (e.g., concatenation, weighted sum, or attention) that combines features from all modalities. Inspired by recent work [373,381], fusion strategies can also include additive fusion mechanisms:

(47) $$\hat{Y}=F_{S}\left(J,B;\theta_{S}\right)+F_{V}\left(V;\theta_{V}\right)+F_{T}\left(T;\theta_{T}\right)$$

where $\hat{Y}$ is the combined prediction from all modalities, $F_{S}\left(\cdot \right)$ processes skeleton joints *J* and RGB frames *B* with parameters $\theta_{S}$, $F_{V}\left(\cdot \right)$ processes video frames *V* with parameters $\theta_{V}$, and $F_{T}\left(\cdot \right)$ processes text data *T* with parameters $\theta_{T}$. A classifier maps the fused features to the final prediction:

(48) $$C\left(\phi_{fuse}\left(X_{i}\right)\right)$$

The per-sample loss is defined as:

(49) $$L\left(C\left(\phi_{fuse}\left(X_{i}\right)\right),y_{i}\right)$$

where $X_{i}$ is the multimodal input for sample *i*, $\phi_{fuse}\left(\cdot \right)$ is the fusion operator applied to extracted features, C(·) is the classifier, and $y_{i}$ is the corresponding ground-truth label.

During training, the average loss over all training samples is computed as:

(50) $$L_{multi}=\frac{1}{n}\sum_{i=1}^{n}L\left(C\left(\phi_{fuse}\left(X_{i}\right)\right),y_{i}\right)$$

where $L_{multi}$ denotes the overall training loss computed over the dataset, *n* is the total number of training samples, $X_{i}$ denotes the input data from all modalities for sample *i*, and $y_{i}$ is the corresponding ground truth label. Recent studies, such as AMFI-Net [372], have proposed advanced attention-based fusion techniques to enhance multimodal integration. AMFI-Net utilizes RGB and skeleton modalities and employs a multi-step fusion strategy.

First, they extracted features from RGB ($X_{Output}^{RGB}$) and skeleton ($X_{Output}^{Skeleton}$) modalities are concatenated:

(51) $$X_{fusion}=\mathrm{Concatenation}\left(X_{Output}^{Skeleton},X_{Output}^{RGB}\right)$$

where $X_{Output}^{RGB}$ is the feature vector from the RGB modality extracted via ResNet3D-18, $X_{Output}^{Skeleton}$ is the feature vector from the skeleton modality extracted via AGCN, and $X_{fusion}$ is the concatenated feature representation combining both modalities. A nonlocal feature association module then captures long-range spatio-temporal dependencies:

(52) $$z_{i}=W_{z}\left(\sum_{\forall j}\frac{e^{\theta {\left(x_{i}\right)}^{T}\beta \left(x_{j}\right)}}{\sum_{\forall j}e^{\theta {\left(x_{i}\right)}^{T}\beta \left(x_{j}\right)}}g\left(x_{j}\right)\right)+x_{i}$$

where $x_{i}$ is the feature vector at position *i* in $X_{fusion}$, θ(·) and β(·) are 1×1 convolution operations computing feature affinities, $g(x_{j})$ is a transformation function (1×1 convolution) applied at position *j*, $W_{z}$ is a learned weight matrix projecting the aggregated nonlocal response, and $z_{i}$ is the enhanced feature vector after applying nonlocal feature association. A channel attention mechanism assigns importance weights to different feature channels:

(53) $$F_{fusion}=\sigma \left(\mathrm{Conv}1D\left(W_{c}Z_{fusion}\right)\right)$$

where $Z_{fusion}$ is the stacked nonlocal feature-enhanced representation for all positions, $W_{c}$ is the learnable weight matrix for the 1D convolution, Conv1D(·) is the 1D convolution operation along the channel dimension, σ(·) denotes the sigmoid activation function, and $F_{fusion}$ is the channel attention weight vector that dynamically emphasizes important features. A dynamic confidence gate for the skeleton modality is defined as:

(54) $$Y_{Skeleton}^{\prime }=\sigma \left(\mathrm{FC}\left(WY_{Skeleton}\right)\right)$$

where $Y_{Skeleton}$ is the skeleton modality feature vector, *W* is a learned weight matrix in the fully connected layer, FC(·) denotes the fully connected layer operation, and $Y_{Skeleton}^{\prime }$ is the dynamic confidence gate output, indicating the importance of the skeleton modality. The final fused features are adaptively weighted:

(55) $$Y_{fused}=Y_{Skeleton}⊙Y_{Skeleton}^{\prime }+Y_{RGB}⊙\left(1-Y_{Skeleton}^{\prime }\right)$$

where $Y_{RGB}$ is the RGB modality feature vector, ⊙ denotes element-wise multiplication, and $Y_{fused}$ is the final adaptively fused feature vector combining both modalities. The overall loss combines classification loss and gating consistency loss:

(56) $$L_{total}=L_{fusion}+\lambda L_{gate}$$

where $L_{fusion}$ is the classification loss (e.g., cross-entropy), $L_{gate}$ is the gating consistency loss enforcing smooth confidence transitions, and λ is the weighting factor balancing the two loss terms. The AMFI-Net model demonstrates superior performance on the NTU RGB+D dataset, achieving a reported accuracy of 95.82% in the multimodal setting (RGB+Skeleton), compared to 93.24% using the skeleton modality alone [372]. This highlights the effectiveness of attention-based multimodal fusion strategies for improving action recognition tasks. Both [381] and [373] highlight the importance of multi-level fusion strategies: weighted ST-ROI enhances key skeleton joints within RGB images, and STDR image construction combines skeleton joints with RGB frames to create dynamic region representations. These strategies improve feature learning by capturing intra- and inter-modal relationships. Different fusion strategies for $\phi_{fuse}\left(\cdot \right)$ include early fusion (input-level concatenation, but can struggle with temporal misalignment), late fusion (decision-level combination, offering modularity but limited feature interactions), hybrid fusion (integrates early and late fusion, balancing flexibility and complexity) [175,381], and attention-based fusion (dynamic weighting, capturing complex dependencies but requiring careful design to avoid overfitting) [373]. Practical challenges include temporal alignment, data imbalance, and increased computational demands. Incorporating semantic information, as in SAM-Net’s semantic assistance module [373], can further improve multimodal learning by enriching feature representations and reducing domain gaps. Additionally, multimodal fusion remains central to advancing HAR performance across complex environments. Table 11 provides a concise comparative analysis, highlighting their integration stages, key advantages, and inherent limitations.

## 7. Current Challenges

Although notable progress has been made in HAR utilising four data modalities, several challenges persist due to the intricate nature of the various aspects of this task.

### 7.1. RGB Data Modality Based Current Challenges

The researcher explores the challenges specific to RGB-based methods in HAR. RGB data, which represents color information from regular images or videos, is widely used for determining human actions. In the following section, we describe the key challenges associated with RGB-based HAR:

#### 7.1.1. Efficient Action Recognition Analysis

The good performance of numerous HAR approaches often comes with the cost of high computational complexity. However, an efficient HAR system is vital for many real-world applications. Therefore, it is essential to explore ways to minimise computational costs (such as CPU, GPU, and energy usage) to perform efficient and fast HAR. These limitations led to a notable impact on the computation efficiency of the network. Additionally, the process of accurately and efficiently labeling video data incurs substantial labor and time expenses due to the diversity and scale of the data.

#### 7.1.2. Complexity Within the Environment

Certain HAR techniques perform strongly in controlled environments but tend to underperform in uncontrolled outdoor settings. This is mostly caused by motion vector noise, which can drastically degrade resolution. Extracting effective features from complex images is an extremely tough task. For example, the rapid movement of the camera complicates the extraction of effective action features. Accurate feature extraction will also affect environmental issues such as poor lighting, dynamic background, etc.

#### 7.1.3. Large Memory of the Dataset and Limitations

The dataset exhibits both intra-class variation and inter-class similarity. Many people perform the same action in diverse manners, and even a single person may execute it in multiple ways. Additionally, different actions might have similar presentations. Furthermore, many existing datasets include unfiltered sequences, potentially compromising the timeliness and reducing the HAR accuracy of the model. The dataset’s large memory requirements pose significant limitations, particularly in terms of storage and processing capabilities. Handling massive amounts of data necessitates robust computational resources, including high-capacity storage solutions and powerful processing units. Additionally, working with large datasets may lead to challenges related to data transfer speeds, memory management, and computational efficiency. These limitations can impact the scalability, accessibility, and usability of the dataset, potentially hindering its widespread adoption and utilization in research and applications. Therefore, addressing the constraints posed by the dataset’s large memory footprint is crucial for maximizing its utility and effectiveness in various domains.

### 7.2. Skeleton Data Modality-Based Challenges

The challenges are specific to skeleton-based approaches in HAR. Skeleton data, which obtain joint positions and movements, are a valuable modality for understanding human actions. In the following section, some key challenges are described.

#### 7.2.1. Pose Preparation and Analysis

Depending on depth cameras and sensors, skeleton data acquisition can be affected by environmental complexity, capture duration, and equipment exposure conditions. Another common challenge in daily-life scenarios is occlusion, caused by surrounding objects or human interaction, which contributes to detection errors. This issue is also discussed in Section 4.2 (Pose Estimation), where occlusion affects the accurate extraction of skeleton points using methods such as MediaPipe, OpenPose, and AlphaPose (see Figure 8). Despite advances in DL-based 2D and 3D pose estimation, occlusion remains a significant challenge in HAR.

#### 7.2.2. Viewpoint Variation

Accurately distinguishing skeleton features from different perspectives poses a significant challenge, as certain features may be lost during changes in viewpoint. Meanwhile, modern RGBD cameras [390,391,392,393] can normalize 3D human skeletons [21,225] from various angles to a single pose with viewpoint invariance, utilizing pose estimation transformation matrices. However, in this process, there is a risk of losing some of the relative motion between the original skeletons. This loss of relative motion can impact the accuracy and completeness of the skeleton data, highlighting the need for careful consideration and validation of viewpoint normalization techniques in skeleton feature extraction.

#### 7.2.3. Single Scale Data Analysis

As several skeleton-based datasets mostly provide information based on the scale of body joints, numerous techniques focus solely on extracting features related to the human joint scale. However, this technique often leads to the loss of fine joint features. Moreover, certain actions, such as shaving, tooth brushing, and applying lipstick, exhibit similar joint interactions. Therefore, there is a critical need to enhance local feature extraction while maintaining the effectiveness of holistic feature extraction techniques [394,395,396,397]. This improvement is crucial for achieving more accurate action recognition and understanding subtle variations in human movements. Even though DL methods yield superior recognition performance compared to handcrafted action features, certain challenges persist in recognizing human actions based on DL, particularly in the fusion of multimodal data in DL methods. Most of the aforementioned DL-based approaches concentrate on learning action features from diverse modality data; however, only a few studies address the fusion of multimodal data. Effective fusion based on multimodal data (RGB, optical flow, depth, and skeleton data) remains a significant unresolved challenge in HAR and DL. This area also represents a prominent research focus within HAR.

### 7.3. Sensor-Based HAR; Current Challenges and Possible Solution

In sensor-based HAR, different activities with similar characteristics (like walking and running) pose a challenge for feature extraction. Creating unique features to represent each activity becomes difficult due to the inter-activity similarity. Another challenge is annotation scarcity due to expensive data collection and class imbalance, particularly for rare or unexpected activities. In sensor-based HAR, three critical factors—users, time, and sensors—contribute to distribution discrepancies between training and test data. These factors include person-dependent activity patterns, evolving activity concepts over time, and diverse sensor configurations. When designing a HAR system, two key considerations are resource efficiency for portable devices and addressing privacy risks associated with continuous life recording. When dealing with sensory data, accurate recognition solutions must address interpretability and understand which parts of the data contribute to recognition and which parts introduce noise. Additionally, we describe radio frequency (RF)-based data for HAR below.

#### Challenges in RF-Based HAR

Despite recent advancements, RF-based HAR continues to face several fundamental challenges due to the complex nature of wireless signal propagation and the absence of structured spatial features typical in vision data:

- Modality Fusion and Latency: Fusing RF signals with data from other modalities (e.g., vision or inertial sensors) introduces challenges such as sampling rate mismatch, latency, and temporal misalignment. Achieving real-time, low-latency fusion while preserving cross-modal synchronization remains an open problem, particularly in mobile or dynamic environments [351,398].
- Representation Learning for RF Signals: Unlike images or video, RF signals lack regular grid-based spatial structure, making direct application of standard deep learning techniques less effective. Developing transferable, domain-invariant representations from complex inputs like CSI, RSSI, or Doppler spectrograms remains a growing area of research [348,399].
- Cross-Domain Generalization: RF-based models often perform poorly when transferred across different environments, hardware setups, or users due to the high sensitivity of wireless signals to ambient changes. While domain adaptation techniques such as adversarial learning and few-shot adaptation have been proposed, robust generalization remains limited relative to vision-based HAR [348,350].
- Multipath Propagation: As discussed in Section 5.4.3, multipath effects arise when signals reflect off surfaces and objects, causing interference patterns that distort amplitude and phase. These distortions degrade the quality of extracted features and the reliability of classification. While mitigation techniques such as Angle-of-Arrival (AoA) estimation, Doppler analysis, and deep domain adaptation have shown promise [340,341], real-time multipath-resilient modelling across diverse spaces remains an unsolved problem.

These open challenges highlight that RF-based HAR is not merely an extension of other modalities but is a distinct and evolving paradigm. It offers compelling advantages such as privacy preservation and through-wall sensing, yet demands novel approaches to signal modelling, robust learning, and scalable deployment.

### 7.4. Multimodal-Based Challenges

In the field of HAR, researchers have explored many multimodal approaches, including fusion-based systems and cross-modality transfer learning. While multimodal fusion can significantly improve HAR performance by leveraging complementary information, several key technical challenges still limit deployment in real-world applications:

#### 7.4.1. Temporal Misalignment

Asynchronous data streams from different sensors can cause misalignment between modalities, complicating effective fusion.

#### 7.4.2. Missing Modalities

In practice, some sensor modalities may be unavailable or corrupted, challenging the robustness of fusion models.

#### 7.4.3. High Computational Cost

Fusion architectures, particularly attention-based or deep-learning models, often demand significant computational resources, limiting real-time or embedded applications.

#### 7.4.4. Overfitting and Heterogeneous Data

Overfitting can occur when models struggle to generalize to new environments. Additionally, different modalities often have heterogeneous formats and temporal scales, complicating integration. These challenges must be addressed to develop more effective and deployable multimodal HAR systems.

## 8. Discussion and Future Direction

In this section, we describe several potential directions for future research by combining the current state of affairs and addressing the methodological and application-related challenges in RGB-based, skeleton-based, sensor modality-based, and multimodal-based HAR. We provide specific implementation pathways and technical considerations to guide researchers toward impactful contributions.

### 8.1. Development of the New Large Scale Datasets

Data are as essential to DL as model construction. However, existing datasets pose challenges when it comes to generalizing to realistic scenes. Factors like realistic surroundings and dataset size play an important role in this complexity. Additionally, most of the datasets are mainly focused on spatial representation [400]. Unfortunately, there is a scarcity of long-term modeling datasets. A notable issue arises due to regional constraints and privacy concerns. As discussed in Section 3.1 (RGB-Based Datasets of HAR), many benchmark datasets such as Kinetics-400 and Kinetics-700 are sourced from YouTube. However, YouTube dataset managers commonly provide only video IDs or links for download rather than the actual video content, resulting in approximately 5% of videos becoming inaccessible annually [28]. To address these limitations, researchers are actively developing new datasets that integrate RGB, depth, and skeleton data to capture multimodal information. These efforts emphasize the need for synchronized data collection, diverse environments, and robust privacy protection measures. Incorporating occlusion scenarios, variable lighting conditions, and long-term temporal sequences is crucial for building realistic and comprehensive benchmarks. Such datasets are expected to contribute significantly to advancing DL research and improving model performance in the future.

### 8.2. Data Augmentation Techniques

Deep neural networks exhibit exceptional performance when trained on diverse datasets. However, limited data availability remains a challenge. To overcome this issue, data augmentation plays an important role. In the domain of image recognition, various augmentation techniques have been proposed, spanning both DL-based techniques and simple image-processing approaches. These approaches include random erasing [401], Generative Adversarial Networks (GANs) [402], kernel filters [403], feature space augmentation [404], adversarial training [405], and meta-learning [406]. For HAR, typical data augmentation techniques involve horizontal flipping, subclip extraction, and video merging [407]. However, these generated videos often lack realism. To overcome this limitation, Zhang et al. [408] used GANs to generate new data samples and implemented a ‘self-paced selection’ strategy during training. Meanwhile, Gowda et al. [409] introduced Learn2Augment, which synthesizes videos from foreground and background sources as a method for data augmentation, resulting in diverse and realistic samples. By implementing these specific recommendations, future research can build more robust data augmentation pipelines that directly address the challenges faced in real-world HAR scenarios.

### 8.3. Advancements in Models Performances

HAR research predominantly revolves around DL-based models, much like other advancements in computer vision. Presently, ongoing progress in deep architectures is important for HAR, including the RGB-based, skeleton-based, and multimodal-based approaches to perform the action recognition task. These advancements typically focus on the following key areas of model improvement:

- Long-term Dependency Analysis: Long-term correlations refer to the sequence of actions that unfold over extended periods, akin to how memories are stored in our brains. In action recognition, it is essential to integrate both spatial and temporal modeling to capture these dependencies. To implement this, future research should consider transformer-based temporal attention models (e.g., Video Swin Transformers), TCNs, and hierarchical recurrent architectures that explicitly model variable-length sequences.
- Multimodal Modeling: This involves integrating data from multiple devices, such as RGB, skeleton, and audio sensors, to build more robust HAR systems. Implementation can leverage cross-modal attention mechanisms (e.g., co-attention modules) to dynamically weight modalities based on scene context. Techniques like cross-modal contrastive learning and domain adaptation can further enhance multimodal fusion performance, addressing occlusion and domain shift issues identified earlier.
- Enhancing Video Representations: Multimodal data (such as depth, skeleton, and RGB) is essential for improving video representations [410,411]. Future research should focus on implementing multi-stream networks, where each stream processes a different modality and shares temporal context through feature fusion layers. Additionally, self-supervised pretraining on unlabeled multimodal videos can improve generalization to unseen environments and handle missing modalities.
- Efficient Modeling Analysis: Creating an efficient network architecture is crucial due to the challenges posed by existing models, including model complexity, excessive parameters, and real-time performance limitations. To address these issues, techniques like distributed training [412], mobile networks [413], hybrid precision training, model compression, quantization, and pruning can be explored. These approaches can enhance both efficiency and effectiveness in image classification tasks.
- Semi-supervised and Unsupervised Learning Approaches: Supervised learning approaches, especially those based on deep learning, typically require large, expensive labeled datasets for model training. In contrast, unsupervised and semi-supervised learning techniques [414] can utilize unlabeled data to train models, thereby reducing the need for extensive labeled datasets. Given that unlabeled action samples are often easier to collect, unsupervised and semi-supervised approaches to HAR represent a crucial research direction deserving further exploration.

By adopting these targeted approaches, future research can systematically address key challenges such as temporal consistency, data scarcity, domain adaptation, and model efficiency, leading to more generalizable and deployable HAR systems.

### 8.4. Video Lengths in Human Action Recognition

The action prediction tasks can be broadly categorized into short-term and long-term predictions. Short-term prediction involves predicting action labels from partially observed actions, typically seen in short videos lasting a few seconds. In contrast, long-term prediction assumes that current actions influence future actions and focuses on longer videos spanning several minutes, simulating changes in actions over time. Formally, given an action video $x_{a}$, which may depict either a complete or incomplete action sequence, the objective is to predict the subsequent action $x_{b}$. These actions, $x_{a}$ and $x_{b}$, are independent yet semantically significant, with a temporal relationship [415]. To advance action prediction research, it is essential to discover and model temporal correlations within vast datasets. Future work should focus on transformer-based and temporal convolutional models to capture long-term dependencies and variable-length sequences. Multimodal fusion of RGB, skeleton, and depth data via cross-modal attention can improve prediction robustness in occluded or cluttered scenes. Additionally, self-supervised learning on unlabelled videos—using temporal consistency regularization and contrastive objectives—can reduce annotation costs and enhance generalization. Finally, interpretability techniques such as temporal attention visualization can help identify the most informative video segments, supporting model transparency and trustworthiness.

#### Limitations

This study focused on research papers published between 2014 and 2025, exclusively in English, and excluded relevant studies in other languages. We exclusively considered studies that utilised visual data, including HAR feature ML-based and DL-based methods involving different data types, including RGB handcrafted features and DL-based action recognition, RGB and skeleton-based methods for multimodal datasets such as RGB, depth, and skeleton, excluding EMG-based data. Furthermore, the diverse input methods and dataset variations across reviewed studies hindered direct result comparisons. Notably, some articles lacked statistical confidence intervals, making it challenging to compare their findings.

## 9. Conclusions

HAR is an important task among multiple domains within the field of computer vision, including human–computer interaction, robotics, surveillance, and security. In the past decades, it has necessitated the proficient comprehension and interpretation of human actions with various data modalities. Researchers still find the HAR task challenging in real scenes due to various complicating factors in different data modalities, including various body positions, motions, and complex background occlusion. In the study, we presented a comprehensive survey of HAR methods, including advancements across various data modalities. We briefly reviewed human action recognition techniques, including hand-crafted features in RGB, skeleton, sensor, and multi-modality fusion with conventional and end-to-end DL-based action representation techniques. Moreover, we have also reviewed the most popular benchmark datasets of the RGB, skeleton, sensor, and fusion-based modalities with the latest performance accuracy. It is worth noting that, although HAR methods have implications in security domains, this survey excludes a focused review on security-related applications. After providing an overview of the literature about each research direction in HAR, the primary effective techniques were presented to familiarize researchers with the relevant research domains. The fundamental findings of this investigation on the study of human action recognition are summarized to help researchers, especially in the field of HAR.

## Figures and Tables

**Table 1:** RGB and deep learning-based existing techniques for action recognition.

Author	Year	Dataset Name	Modality	Method	Classifier	Accuracy [%]
Ji et al. [46]	2012	KTH	RGB	3DCNN		90.2
Wang et al. [47]	2015	UCF101 HMDB51	RGB	2-stream Convolution Network	Softmax	91.5 65.9
Sharma et al. [48]	2015	UCF11 HMDB51 Hollywood2	RGB	Stacked LSTM	Softmax	84.96 41.31 43.91
Ijjina et al. [49]	2016	UCF50	RGB	CNN-Genetic Algorithm	CNN	99.98
Feichtenhofer et al. [50]	2016	UCF101 HMDB51	RGB	CNN Two-Stream	Softmax	92.5 65.4
Wang et al. [51]	2016	HMDB51 UCF101	RGB	TSN	Softmax	69.4 94.2
Akilan et al. [52]	2017	CIFAR100 Caltech101 CIFAR10	RGB	ConvNets	Softmax	75.87 95.54 91.83
Shi et al. [53]	2017	KTH UCF101 HMDB51	RGB	3-stream CNN	Softmax	96.8 94.33 92.2
Ahsan et al. [54]	2018	UCF101 HMDB51	RGB	GAN	Softmax	47.2 41.40
Tu et al. [55]	2018	JHMDB HMDB51 UCF Sports UCF101	RGB	Multi-Stream CNN	Softmax	71.17 69.8 58.12 94.5
Zhou et al. [56]	2018	HMDB51 UCF101	RGB	TMiCT-Net	CNN	70.5 94.7
Jian et al. [57]	2019	Sport video	RGB	FCN	Softmax	97.40
Ullah et al. [44]	2019	UCF50 UCF101 YouTube action HMDB51	RGB	Deep autoencoder	SVM	96.4 94.33 96.21 70.33
Gowda et al. [58]	2020	UCF101 HMDB51 FCVID ActivityNet	RGB	SMART	Softmax	98.6 84.3 82.1 84.4
Khan et al. [59]	2020	HMDB51 UCF Sports YouTube IXMAS KTH	RGB	VGG19 CNN	Naive Bayes	93.7 98.0 94.4 99.4 95.2 97.0
Ullah et al. [60]	2021	HMDB51 UCF101 UCF50 Hollywood2 YouTube Actions	RGB	DS-GRU	Softmax	72.3 95.5 95.2 71.3 97.17
Wang et al. [61]	2021	SomethingV1 SomethingV2 Kinetics-400	RGB	Temporal Difference Networks	TDN	84.1 91.6 94.4
Wang et al. [62]	2022	UCF101	RGB	HyRSM	-	93.0
Wensel et al. [63]	2023	YouTube Action HMDB51 UCF50 UCF101	RGB	ViT-ReT	Softmax	92.4 78.4 97.1 94.7
Hassan et al. [64]	2024	UCF11 UCF Sports JHMDB	RGB	Deep Bi-LSTM	Softmax	99.2 93.3 76.3
Khan et al. [65]	2025	UCF50 HMDB51 UCF101	RGB	ConvLSTM and LRCN	Softmax	97.42 73.63 95.70
Shah et al. [66]	2025	UCF101 HMDB51	RGB	KD-GAN	Softmax	98.50 79.21

**Table 3:** Action recognition methods based on handcrafted feature extraction techniques.

Author	Year	Dataset Name	Modality	Method	Classifier	Accuracy [%]
Gan et al. [112]	2013	UTKinect-Action	RGB	RF	APJ3D	92.00
Everts et al. [113]	2014	UCF11 UCF50	RGB	Multi-channel STIP	SVM	78.6 72.9
Zhu et al. [114]	2014	MSRAction3D UTKinectAction CAD-60 MSRDailyActivity3D HMDB51	RGB	STIP (HOG/HOF)	SVM	94.3 91.9 87.5 80.0
Yang et al. [21]	2014	MSR Action3D	RGB	EigenJoints-based	NBNN	97.8
Liu et al. [115]	2015	KTH HMDB51 UCF YouTube Hollywood2	RGB	GP-learned descriptors	SVM	95.0 48.4 82.3 46.8
Xu et al. [116]	2016	MSRAction3D UTKinectAction Florence 3D-Action	RGB	PSO-SVM	-	93.75 97.45 91.20
Vishwakarma et al. [117]	2016	KTH Weizmann i3Dpost Ballet IXMAS	RGB	SDEG	SVM	95.5 100 92.92 93.25 85.8
Singh et al. [118]	2017	UCSDped-1 UCSDped-2 UMN	RGB	Graph formulation	SVM	97.14 90.13 95.24
Jalal et al. [119]	2017	IM-DailyDepthActivity MSRAction3D MSRDailyActivity3D	RGB	HOG-DDS	HMM	72.86 93.3 97.9
Nazir et al. [120]	2018	KTH UCF Sports UCF11 Hollywood	RGB	D-STBoE	SVM	91.82 94.00 94.00 68.10
Ullah et al. [121]	2021	UCF Sports UCF101	RGB	Weekly supervised based	SVM	98.27 84.72
Al et al. [122]	2021	E-KTH E-UCF11 E-HMDB51 E-UCF50 R-UCF11 R-UCF50 N-Actions	RGB	Local and global feature extraction	QSVM	93.14 94.43 87.61 69.45 82.61 68.96 61.94
Hejazi et al. [123]	2022	UCF101 Kinetics-400 Kinetics-700	RGB	Optical flow based	KNN	99.21 98.24 96.35
Zhang et al. [124]	2022	UCF 11 UCF 50 UCF 101 JHMDB51 UT-Interaction	RGB	FV+BoTF	SVM	99.21 92.5 95.1 70.8 91.50
Fatima et al. [125]	2023	UT-Interaction	RGB	SIFT and ORB	Decision Tree	94.6

**Table 5:** Comparison of prominent GCN-based models used in HAR.

Model	Innovation Point	Strengths	Limitations
ST-GCN [244]	Fixed skeleton graph structure with spatial–temporal convolution	Baseline for spatial–temporal modeling	Limited flexibility for unseen poses and graph variation
2s-AGCN [201]	Data-driven topology learning and attention-based weighting	Improved adaptability and robustness	High computational complexity; sensitivity to sensor noise
STA-GCN [245]	Attentional focus on action-relevant joints and frames	Enhanced interpretability; adaptive attention	Requires careful tuning of attention mechanisms
Shift-GCN [246]	Spatial shift operations for efficient receptive field expansion	Lightweight and efficient; good for long actions	Less expressive than full GCN in small-scale motions
InfoGCN [252]	Injects global semantics into GCN to improve feature learning	Improved generalization; handles complex scenes	May require large training data for stable learning
EMS-TAGCN [217]	Multi-stream adaptive attention across space, time, and channels	High accuracy across datasets; modular attention mechanism	Increased complexity; scalability concerns without further tuning

**Table 8:** Multi-modalitydata fusion-based HAR system models and their performance metrics.

Dataset	Classifier	Methods	Data Set Type	Year	Reference	Accuracy [%]
NTU RGB+D (CS) NTU RGB+D (CV)	SVM	P-LSTM	RGB, Depth	2016	[91]	62.93 70.27
UCI-HAD USC-HAD Opportunity Daphnet FOG Skoda	SVM KNN	DRNN	Sensors	2017	[330]	96.7 97.8 92.5 94.1 92.6
Smartwach	Softmax	Dilated CNN	Sensor	2020	[300]	95.49
UTD-MHAD NTU RGB+D	Softmax	Vission based	RGB, Depth, Skeleton	2021	[363]	98.88 75.50
NTU RGB+D (CS) NTU RGB+D (CV) SYSU 3D HOI UWA3D II	Hierarchical- score fusion	Multi Model	RGB Depth	2021	[364]	89.70 92.97 87.08
UCF-101 Something-Something-v2 Kinetics-600	Softmax	MM-ViT	RGB	2022	[365]	98.9 90.8 96.8
MHEALTH UCI-HAR	Softmax	CNN-LSTM	Sensor	2022	[366]	98.76 93.11
UCI-HAR WISDM MHEALTH PAMAP2 HHAR	SVM	CNN with GA	Sensors	2023	[305]	98.74 98.34 99.72 97.55 96.87
NTU RGB+D 60 NTU RGB+D120 PKU-MMD Northwestern UCLAMultiview Toyota Smarthome	-	MMNet	RGB, Depth	2023	[367]	98.0 90.5 98.0 93.3
NTU RGB+D 60 NTU RGB+D120 NW-UCLA	Softmax	InfoGCN	RGB, Depth	2023	[252]	93.0 89.8 97.0
NTU RGB+D NTU RGB+D120	Softmax	Two-stream Transformer	RGB, Depth	2023	[368]	94.8 93.8
NTU RGB+D NTU RGB+D120 NW-UCLA	Softmax	Language knowledge-assisted	RGB, Depth	2023	[369]	97.2 91.8 97.6
UCF51 Kinetics Sound	Softmax	MAIVAR-CH	RGB, audio	2024	[370]	87.9 79.0
Drive Act	-	Dual Feature Shift	RGB, Depth, Infrared	2024	[99]	77.61
Florence3DAction UTKinect-Action3D 3DActionPairs NTURGB+D	Softmax	two-stream spatial–temporal architecture	RGB, Depth, Infrared	2024	[371]	93.8 98.7 97.3 90.2
UI-PRMD KIMORE	Softmax	Fusing CNNs	RGB, Skeleton	2025	[360]	89.80 95.33
Custom HAR	Softmax	Multi-Features Fusion CNN	Sensor	2025	[361]	97.92
Custom Gymnastics Activity UCI-HAR	Softmax	CIR-DFENet	Sensor	2025	[362]	99.40 98.07
OPPT PAMAP2 DSADS	Softmax	DTSDA	Sensor	2025	[327]	99.00 81.55 51.59
NTU-RGB+D UTD-MHAD	Softmax	AMFI-Net	RGB Skeleton	2025	[372]	88.97 93.21
NTU-60 PKU-MMD Northwestern UCLA	Softmax	SAM-Net	RGB, Skeleton, text	2025	[373]	94.8 97.0 93.7

**Table 10:** Modality-based unified dataset comparison for the multimodal dataset work.

Algorithm	Dataset Name	Skeleton	RGB	Depth	Sensor Signal	Others	Modality Summary	Acc.	Prec.	Rec.
Khaire et al. [377]	NTU RGB +D	-	RGB	-	-		Single	70.01%	-	-
Khaire et al. [377]	NTU RGB +D	Skelton	-	-	-		Single	69.90%	-	-
Khaire et al. [377]	NTU RGB +D	-	-	Depth	-	-	Single	80.30%	-	-
Khaire et al. [377]	NTU RGB +D	-	RGB	Depth	-	-	Multi	91.16%	-	-
Khaire et al. [377]	NTU RGB +D	Skeleton	RGB	-	-	-	Multi	80.69%	-	-
Khaire et al. [377]	NTU RGB +D	Skeleton	-	Depth	-	-	Multi	93.50%	-	-
Khaire et al. [377]	NTU RGB +D	Skeleton	RGB	Depth	-	-	Multi	94.60%	-	-
Zhao et al. [372]	NTU RGB +D	-	RGB	-	-		Single	95.22%		
Zhao et al. [372]	NTU RGB +D	Skeleton	-	-	-		Single	93.24%	-	
Zhao et al. [372]	NTU RGB +D	Skeleton	RGB	-	-		Multi	95.82%	-	
Zhao et al. [372]	UTD-MHAD	Skeleton	RGB	-	-	-	Multi	93.21%	-	
Franco et al. [382]	CAD-60, CAD-120, OAD	-	RGB	-	-	-	Single	-	92.5, 61.1, 85.8	89.4, 59.3, 85.9
Franco et al. [382]	CAD-60, CAD-120, OAD	Skeleton	-	-	-	-	Single	-	95.00, 77.60, 80.6	95.0, 73.1, 80.5
Franco et al. [382]	CAD-60, CAD-120, OAD	Skeleton	RGB	-	-	-	Multi	-	98.8, 85.4, 90.6	98.3, 83.3, 90.4
Shah et al. [383]	NTU-60, N-UCLA	-	RGB	-	-	-	Single	98.0, 91.7	-	-
Wu et al. [384]	NTU-60, N-UCLA	Skeleton	-	-	-	-	Single	96.7, 96.8,	-	-
Liu et al. [381]	NTU-60, PKU-MMD, N-UCLA	Skeleton	RGB	-	-	-	Multi	98.0, 98.0, 90.8	-	-
Liu et al. [373]	NTU-60, PKU-MMD, N-UCLA	Skeleton	RGB	-	-	Text	Multi	98.5, 98.4, 92.3	-	-

**Table 11:** Comparison of multimodal fusion strategies in HAR.

Fusion Type	Fusion Stage	Key Advantages	Key Limitations
Early Fusion [377]	Input or low-level feature stage	Simple structure; captures low-level dependencies	Temporal misalignment; feature redundancy
Late Fusion [378]	Output or decision stage	Robust to missing modalities; modular	Ignores feature interaction; limited synergy
Hybrid Fusion [379]	Combines low- and high-level fusion	Improved flexibility; hierarchical modeling	Complex training; higher resource cost
Attention-Based Fusion [367,368,386]	Feature-wise dynamic weighting	Learns modality importance adaptively; robust under occlusion	Overfitting risk; needs large labeled datasets

## Data Availability

No new data were created or analyzed in this study.

## Abbreviations

HAR	Human Activity Recognitions
HOF	Histogram of Optical Flow
STIP	Spatio-Temporal Interest Point
GCN	Graph Convolutional Network
MEI	Motion Energy Image
SVM	Support Vector Machine
TSN	Temporal Segment Network
RF	Radio Frequency
ML	Machine learning
KNN	K-Nearest-Neighbor
CNN	Convolutional Neural Network
DL	Deep Learning
LSTM	Long Short Term Memory
DNNs	Deep Neural Networks
